# Tumor angiogenesis: causes, consequences, challenges and opportunities

**DOI:** 10.1007/s00018-019-03351-7

**Published:** 2019-11-06

**Authors:** Roberta Lugano, Mohanraj Ramachandran, Anna Dimberg

**Affiliations:** The Rudbeck Laboratory, Department of Immunology, Genetics and Pathology, Uppsala University, 75185 Uppsala, Sweden

**Keywords:** Angiogenesis, Cancer, Endothelial, Vascular targeting, VEGF, Anti-angiogenic therapy

## Abstract

Tumor vascularization occurs through several distinct biological processes, which not only vary between tumor type and anatomic location, but also occur simultaneously within the same cancer tissue. These processes are orchestrated by a range of secreted factors and signaling pathways and can involve participation of non-endothelial cells, such as progenitors or cancer stem cells. Anti-angiogenic therapies using either antibodies or tyrosine kinase inhibitors have been approved to treat several types of cancer. However, the benefit of treatment has so far been modest, some patients not responding at all and others acquiring resistance. It is becoming increasingly clear that blocking tumors from accessing the circulation is not an easy task to accomplish. Tumor vessel functionality and gene expression often differ vastly when comparing different cancer subtypes, and vessel phenotype can be markedly heterogeneous within a single tumor. Here, we summarize the current understanding of cellular and molecular mechanisms involved in tumor angiogenesis and discuss challenges and opportunities associated with vascular targeting.

## Introduction

Malignant cells require oxygen and nutrients to survive and proliferate, and therefore need to reside in close proximity to blood vessels to access the blood circulation system. The early observation that rapidly growing tumors were heavily vascularized, while dormant ones were not, led Judah Folkman to propose that initiation of tumor angiogenesis was required for tumor progression [1]. Further, Folkman isolated a tumor-derived factor that induced angiogenesis [2] and hypothesized that inhibition of angiogenic signaling pathways might block new vessel formation and result in tumor dormancy. This exciting concept attracted considerable interest from the research community and spurred extensive efforts dedicated to isolating tumor-derived pro-angiogenic factors and delineating their signaling pathways [3]. In 2003, a clinical trial demonstrating prolonged survival of patients with metastatic colorectal cancer when chemotherapy was administrated in combination with humanized neutralizing antibodies targeting anti-vascular endothelial growth factor (VEGF) resulted in an FDA approval and provided proof-of-concept that anti-angiogenic therapy can be successfully used to treat cancer [4]. Subsequently, several antibodies and tyrosine kinase inhibitors designed to target pro-angiogenic signaling have been approved as cancer therapies. Despite the ever-growing list of FDA-approved drugs, the success of anti-angiogenic therapy has so far been quite limited, only providing short-term relief from tumor growth before resistance occurs and typically resulting in modest survival benefits. The limited efficacy has several explanations including tumors employing alternative modes of angiogenesis and development of resistance mechanisms. In addition, many tumors can obtain access to blood supply through vascular co-option, bypassing the need of tumor angiogenesis [5]. In this review, we summarize the current understanding of molecular and cellular mechanisms involved in tumor angiogenesis, the molecular and functional heterogeneities of tumor vessels and emerging concepts for vascular targeting during cancer therapy.

## Initiation of tumor vascularization: the angiogenic switch

Small dormant tumors that are devoid of active blood vessel formation can frequently be observed in human tissue and in genetically engineered mouse models of multistage carcinoma at early stages of cancer progression. Tumor progression is often accompanied by ingrowth of blood vessels, consistent with a need for malignant cells to have access to the circulation system to thrive. Tumors can be vascularized either through co-option of the pre-existing vasculature [5], or by inducing new blood vessel formation through a variety of molecular and cellular mechanisms briefly described below. Vascular homeostasis is regulated by a large number of pro- and anti-angiogenic factors. When these are in balance, the vasculature is quiescent and endothelial cells are non-proliferative. Initiation of blood vessel formation is induced when pro-angiogenic signaling is dominating, a process that in tumors has been coined the “angiogenic switch” [6]. The angiogenic switch releases tumors from dormancy and sparks rapid growth of malignant cells in association with new blood vessel formation. The development of genetically engineered mice modelling multistage tumor progression has been instrumental in investigating the angiogenic switch. One of the most widely studied models is the RIP1-Tag2 model of pancreatic insulinoma expressing the semian virus 40 large T (SV40T) oncogene under the rat insulin promoter, which was developed in Douglas Hanahan’s laboratory [7]. In this model, tumors develop sequentially in mice carrying the transgene, initiating as non-angiogenic clusters of dysplastic cells, of which a proportion later develop to small angiogenic tumor islets that can progress to large vascularized tumors that metastasize to the lung. By combining this and other murine tumor models with advanced in vitro and in vivo models of angiogenesis [8], a wide range of factors and cellular mechanisms have been described that can initiate vessel formation in tumors. The angiogenic switch can be triggered either by additional genetic alterations of tumor cells, leading to increased proliferation and hypoxia or expression of pro-angiogenic factors, or by tumor-associated inflammation and recruitment of immune cells.

## Mechanisms of blood vessel formation in tumors

**Fig. 1:**
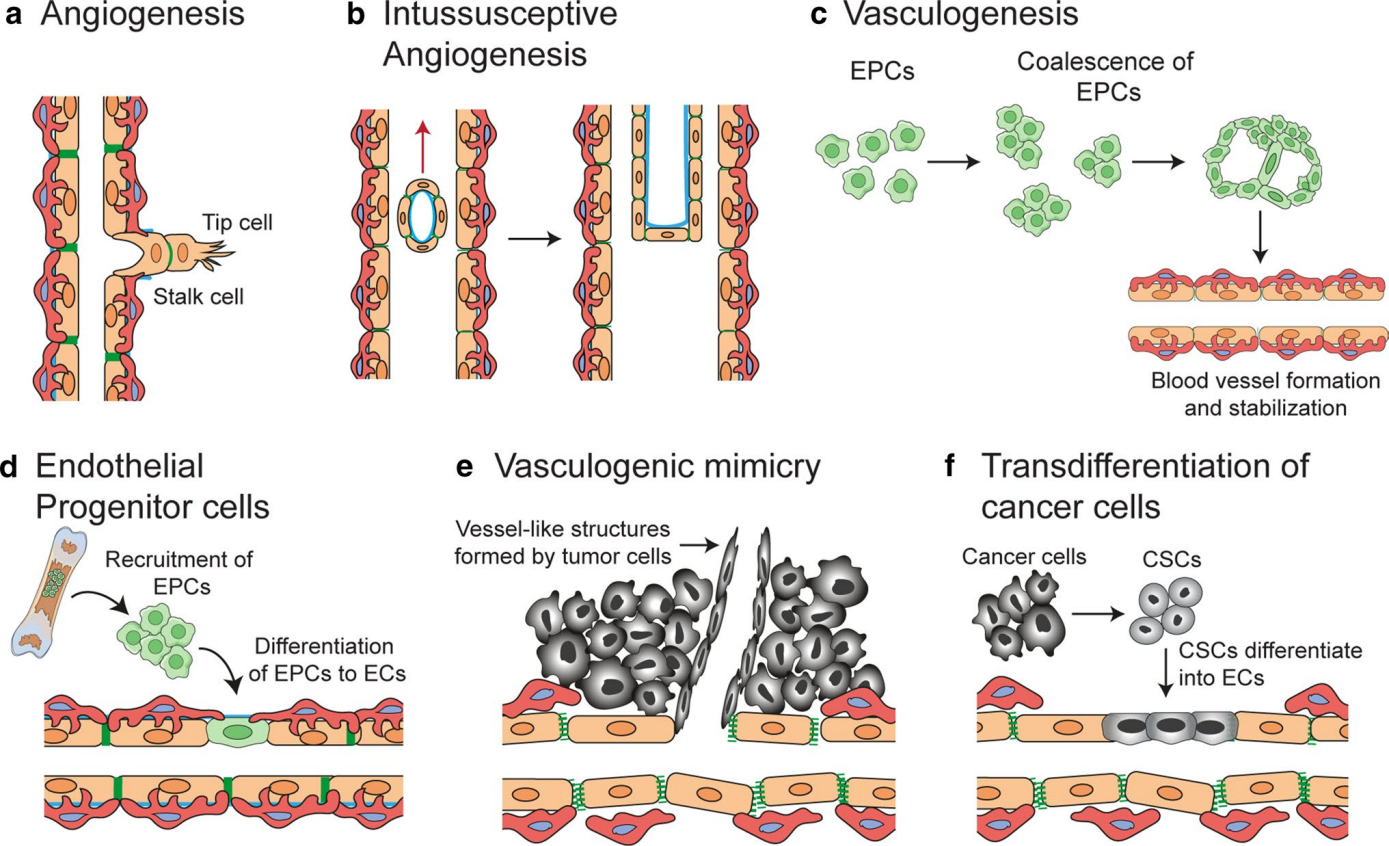
Mechanisms of blood vessel formation. Neo-vascularization in normal tissues and tumors occur through one or more of the following mechanisms: **a** Sprouting angiogenesis: a process involving formation and outgrowth of sprouts (tip cells), which eventually fuse with an existing vessel or newly formed sprout. **b** Intussusceptive angiogenesis: the formation of new vasculature where a pre-existing vessel splits in two. **c** Vasculogenesis: prenatal neo-vascularization from endothelial progenitor cells. The endothelial progenitor cells proliferate and form lumens, eventually assembling into new blood vessels. **d** Recruitment of endothelial progenitor cells: vessel formation in tumors by recruitment of circulating endothelial progenitor cells. **e** Vascular mimicry: a matrix-embedded fluid-conducting meshwork formed by tumor cells. **f** Trans-differentiation of cancer stem cells (CSC): neo-vascularization in tumors through differentiation of CSCs to endothelial cells

The blood circulation system is critical in delivering nutrients and chemicals to tissues, removing waste products, and maintaining homeostasis. The vascular system, composed of the aorta, arteries, capillaries and veins transports blood throughout the body. Arteries carry blood away from the heart, transporting oxygenated blood to the tissues. The capillary networks have narrow walls that help in gas exchange between the blood and tissues. Oxygen is released into the tissues and carbon dioxide is absorbed by the blood, and is transported back to the heart through veins. Transmigration of immune cells into tissues is facilitated by post-capillary venules. The capillary wall is made of an endothelial cell layer surrounded by a basement membrane and is supported by pericytes. Angiogenesis is typically initiated from the capillaries and it plays an important part in tumor growth, maintenance and metastasis. Blood vessel formation in tumors can be induced through several cellular processes (Fig. 1) as briefly summarized below.

### Sprouting angiogenesis

New capillaries can bud from parental vessels through a multi-step process known as sprouting angiogenesis. Formation of sprouts involves (a) Tip cell selection: a cell from the parent vessel becomes the migratory leading cell and blocks its neighboring cells from adopting a tip cell fate by a lateral inhibition process. (b) Sprout extension: the tip cell migrates along the chemotactic path, followed by trailing stalk cells and (c) Lumen formation: connection of the luminal space of the sprout with the parent vessel. The developing sprout then connects with other vessels through a process called anastomosis.

Endothelial cells are normally quiescent, but can be induced to sprout and initiate angiogenesis by pro-angiogenic factors including vascular endothelial growth factor (VEGF). Tip and stalk cell selection is regulated through cross-talk between the VEGF and Dll4/Notch pathways [9]. In response to VEGF, tip cells produce Delta-like-4 (DLL4) ligand, platelet derived growth factor-B (PDGF-B), VEGF receptor-2 (VEGFR-2) and VEGFR-3/Flt-4 [10–12]. VEGF blocks Notch signaling and enhance sprouting, branching, migratory capacity and filopodia formation in tip cells [13]. DLL4 secreted by tip cells activated Notch signaling in the neighboring endothelial cells, suppressing tip cell formation by inhibiting VEGFR2 and VEGFR3 expression and inducing VEGFR1 (decoy for VEGF) expression [14–17]. Tip cells extend numerous filopodia, and acquire motile and invasive phenotypes, secreting matrix degrading proteins that guide new blood vessel formation towards the VEGF gradient [18]. Neuropilins, which are non-tyrosine kinase receptors, promote tip cell function by enhancing signaling through VEGFR2 and VEGFR3 [19, 20]. Stalk cells follow the tip cells and branch out from the parent vessel, establishing the vascular lumen and junctional connections to the forming sprout. They are more proliferative and have fewer filopodia as compared to the tip cells, a process fine-tuned by Notch-regulated ankyrin repeat protein [21]. The term vascular anastomosis defines development of junction between two new sprouts (‘head-to-head’ anastomosis) or a sprout and an existing blood vessel (‘head-to-side’ anastomosis). Live imaging studies in Zebrafish indicate that development of cell junctions is a highly stereotypical process [22–24]. Migrating tip cell filopodia express junctional proteins such as VE-cadherin [25, 26]. The filopodia from adjacent tip cells make and break contacts many times during initial contact formation, after which VE-cadherin is deposited at a single point of filopodia contact and a ring shaped junction is formed to create a small luminal pocket at this site [22, 25, 27]. Next, the excess filopodia retract, membranes of the anastomosing fuse, express apical markers like podocalyxin, and upregulate expression of junctional molecules on the cell surface [25]. The different mechanisms by which the lumen and perfused tubes form are termed type I and type II anastomosis, reviewed in detail by Betz et al. [28].

### Intussusceptive angiogenesis

A less studied process of neo-angiogenesis is “intussusception”, where transluminal tissue pillars develop within existing vessels and subsequently fuse to remodel the vascular plexus, first described in remodeling of lung capillaries [29, 30]. The molecular mechanisms involved in intussusceptive angiogenesis are not completely understood, but the process can be induced by growth factors including VEGF, PDGF and erythropoietin [31–33]. Intussusceptive angiogenesis have been observed in various tumor types including melanoma, colorectal cancer, glioma and mammary tumors [34–37]. In melanoma, VEGF expression correlates with the occurrence of intussusceptive angiogenesis and the number of intraluminal tissue folds [34]. Xenografts of human adenocarcinoma utilize intussusceptive angiogenesis as a mode for rapid vascular remodeling and maintenance of blood flow in tumors [36]. Intususceptive angiogenesis is thought to contribute to tumor growth by increasing the complexity and number of microvascular structures within the tumor.

### Vasculogenesis and recruitment of endothelial progenitor cells

De novo blood vessel formation in the embryo is induced through differentiation and association of endothelial progenitor cells (EPCs) in a process coined vasculogenesis [38, 39]. In mice, progenitor cells differentiate and assemble into clusters called blood islands, as early as embryonic day (E) 6.5–7 [40]. A subset of cells located at the perimeter of the blood islands, termed angioblasts give rise to precursors for endothelial cells, while those at the center differentiate to hematopoietic cells. Angioblasts migrate to the paraxial mesoderm, assemble into aggregates, proliferate and differentiate to form a plexus with endocardial tubes in mouse. This leads to formation of dorsal aortae, cardinal veins and the embryonic stems of arteries and veins in the yolk sac. Vasculogenesis is also described in adults during capillary formation post ischemia [41] or in tumors as alternative mechanism for neo-vascularization to meet the increasing need for oxygen and nutrient supply [42]. In preclinical glioma models, it has been shown that revascularization that occurs during glioma recurrence after irradiation is mediated by vasculogenesis and not angiogenesis [43]. Vasculogenesis in tumors is mediated by recruitment of EPCs or bone marrow–derived hematopoietic cells, resulting in the formation of new vessels to support tumor growth [44, 45]. EPCs are mostly unipotent adult stem cells that have the capacity to self-renew, proliferate, take part in neovascularization and repair endothelial tissue [46, 47]. They were first identified in 1997 by Asahara et al. [41]. EPCs are characterized by expression of CD34, VEGFR1, CD133, Tie-2 (endothelial receptor tyrosine kinase), Nanog and Oct-4 (Octamer-4), and by their ability to bind Ulex-lectin and uptake acetylated low-density lipoproteins [48, 49]. EPCs can be derived from hematopoietic stem cells, myeloid cells, circulating mature endothelial cells or other circulating progenitor cells [46, 50]. EPCs contribute to postnatal vasculogenesis, and are recruited from the bone marrow to sites of injury via growth factors, cytokines and hypoxia-related signaling pathways, where they differentiate into mature endothelial cells and incorporate themselves into sites of active neovascularization [41, 51]. In tumors, vasculogenesis is initiated by crosstalk between tumor cells and EPCs in the bone marrow. VEGF in the tumor microenvironment mobilizes VEGFR2^+^ EPCs from the bone marrow [52–54]. Tumors also secrete other factors well known to mobilize EPCs to the tumor bed and promote neovascularization, including chemokines C–C motif ligand (CCL)2 and CCL5, the hypoxia responsive chemokine CXCL12 (also known as SDF-1) [55] and adiponectin [55–57].

### Vascular mimicry

Aggressively growing tumor cells can form vessel like structures through a process denoted as vascular mimicry. These structures, which are formed without contribution of endothelial cells, represents an alternate channel for tumor cells to source sufficient blood supply and nutrients. Vascular mimicry has been observed in many tumor types including melanoma [58], glioma [59], head and neck cancer [60], lung cancer [61], colorectal cancer [62] and prostate cancer [63]. The existence and relative importance of vascular mimicry was initially debated and questioned in the field [64], but has since been supported by findings of several research groups [65]. Structures formed through vascular mimicry are identified in tumor samples with IHC using CD31 and periodic acid–Schiff (PAS) as markers [66]. The endothelial-like tumor cells can secrete collagens IV and VI, proteoglycans, heparan sulfate, laminin and tissue transglutaminase antigen 2, aiding in tubular structure formation and stabilization [67]. Tumor cells participating in vascular mimicry in uveal melanoma have a multipotent, stem cell-like phenotype and express CD271 [68]. Both vascular mimicry and fibrovascular septa are present in the stroma of melanoma and can be distinguished by their thickness and lamination [69].

Vascular mimicry can contribute to tumor progression in several ways. In melanoma, mitochondrial reactive oxygen species induce activation of the Met proto-oncogene under hypoxic conditions, promoting vascular mimicry. This results in tumor cell motility, invasion, and metastasis [70]. In gliomas, increased vascular mimicry has been reported following anti angiogenic therapy [71]. This may serve as an alternative neovascularization process adopted by the tumor to cope with the therapy and counteract the hypoxic environment. Vascular mimicry is a marker for poor prognosis in several cancer types [62, 72]. However, there is a lack of techniques that can be used to clearly distinguish vascular mimicry from normal endothelial cell lining, which hampers investigations of the relative importance of this process.

### Trans-differentiation of cancer stem cells

Trans-differentiation of cancer stem cells to endothelial cells and vascular smooth muscle-like cells, giving rise to neovascularization, has been reported in several tumor types [59, 73–76]. Tumor endothelial cells have in some studies been observed to harbor similar somatic mutations as the malignant cells of the tumor, indicating a neoplastic origin [59, 73]. Trans-differentiation of glioma cells to endothelial cells in vitro was demonstrated by culturing of glioma cancer stem cells in endothelial-promoting media, resulting in expression of pan-endothelial markers CD31, CD34 and vWF, formation of tubular structures and uptake of LDL [59, 77]. In vivo xenografts of human glioma stem cells were observed to develop tumor vessels with endothelial cells expressing human endothelial proteins CD34, CD144, and VEGFR2. Selective therapeutic targeting of tumor-derived cells expressing Tie-2 could disrupt the vasculature and eradicate the tumor, leading the authors to conclude that glioma stem cell derived endothelial cells contributed to vascularization of glioma [59]. However, these results have been controversial and the clinical relevance has been questioned since endothelial cells in human glioblastoma have not been observed to harbor genetic alterations in other studies [78, 79]. Notably, Tie-2 is not only a marker of endothelial cells, but is also expressed by proangiogenic monocytes and pericytes [80]. A later study using lineage-specific fluorescent reporters did not support tumor cells as a source of endothelial cells in glioma, instead demonstrating that glioma cancer stem cells can differentiate into pericytes and that specific depletion of pericytes disrupted tumor vessels and tumor growth [81]. The trans-differentiation of glioma cancer stem cells to pericytes was enhanced by TGFβ, and their recruitment to endothelial cells was mediated by CXCL12/CXCR4 signaling [81].

## Molecular and functional features of tumor blood vessels

**Fig. 2:**
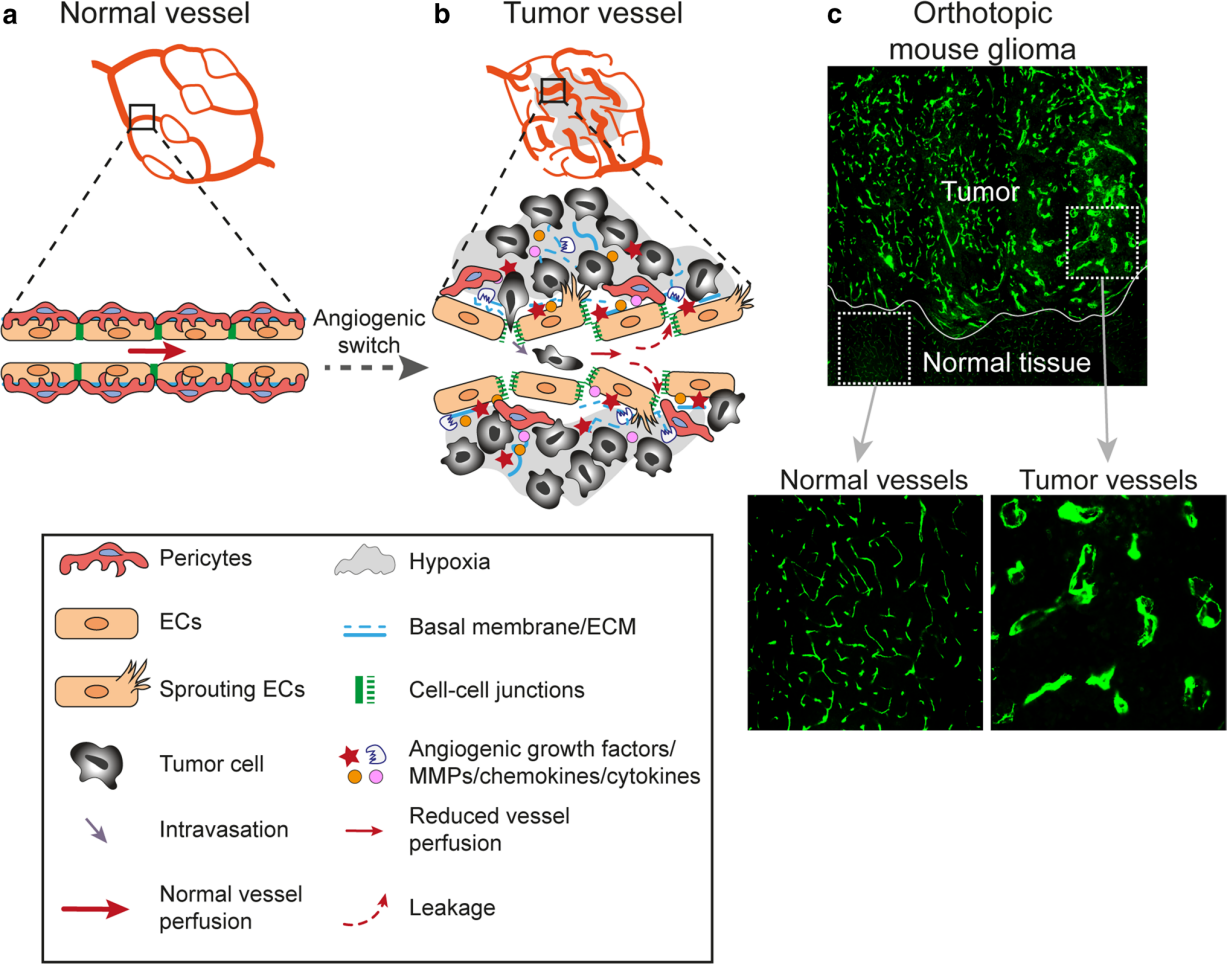
Morphological and functional characteristics of tumor vessels as compared to normal vessels. **a** Normal vessels display an organized and hierarchical branching pattern of arteries, veins, and capillaries. In healthy vessels, endothelial cells are supported by basal membrane and pericytes coverage and they are tightly connected by stable cell-cell junctions. **b** Tumor vessels are morphologically and functionally different from normal vessels. In response to persistent and imbalanced expression of angiogenic factors and inhibitors, tumor vessels display an unorganized network lacking of a hierarchical vessel division. Tumor vessels are characterized by reduced blood flow, endothelial cell sprouting, disruption of endothelial cell junctions, loss of pericytes coverage and increased vessel leakiness resulting in increased tissue hypoxia and intravasation of tumor cells. Moreover, tumor endothelial cell basal membrane is abnormal, including loose associations with endothelial cells and variable thickness. **c** Tumor vessel abnormalization shown by immunofluorescent staining for the vessel marker CD31 (green) in an orthotopic syngeneic mouse model of glioma growing in the brain

While physiological blood vessels formation occurring during development, menstrual cycle or wound healing is a tightly controlled process that ceases when the need for new blood vessels have been met, tumor angiogenesis is deregulated due to a persistence of pro-angiogenic factors in the tumor microenvironment. Efficient circulation depends on an ordered division of the vascular tree into arteries, arterioles, capillaries, venules and veins. However, in the presence of constant pro-angiogenic signaling in the tumor, the newly formed vascular networks may fail to mature and prune, the division into arterioles, capillaries and venules may be lacking, vessel caliber size can be markedly heterogeneous and blood flow through the poorly organized and malformed vessels can be chaotic [82, 83]. This can lead to uneven blood flow within the tumor parenchyma resulting in areas of persisting or intermittent hypoxia [84, 85]. Endothelial junctions are often disrupted in tumor vessels, leading to enhanced permeability, and interstitial fluid pressure is increased [86]. This can in turn reduce the efficacy of cancer therapy since compression of tumor vessels and poor vascular perfusion hamper drug delivery [87]. Pericytes are generally partially detached from endothelial cells in tumor vessels, and the basement membrane is unevenly distributed, leading to increased vessel fragility and risks of hemorrhage [88–90]. Defects in vascular function and integrity profoundly alters the tumor microenvironment (Fig. 2a–c). However, the extent of structural and functional abnormalities observed in tumor vessels vary greatly depending on the tumor type and anatomical location, and also within the same tumor depending on the tumor microenvironment.

Aside from the structural and functional defects observed within the tumor vasculature, tumor blood vessels are molecularly distinct from normal vessels since they respond to environmental cues by transcriptional regulation of gene expression [91–99]. Transcriptional signatures of tumor endothelial cells may vary depending on the anatomic location, the tumor type and the malignancy grade. However, tumor vessels typically up-regulate subsets of genes that are transcriptionally active also during developmental and physiological angiogenesis. Consistent with this, a meta-analysis of transcriptional profiles from different types of human cancer identified a core gene signature including, e.g. VEGFR2, TIE1 and TIE2 which are central regulators of pro-angiogenic VEGF and angiopoietin signaling [100]. This tumor angiogenesis core gene signature also included CLEC14A and CD93, which together with endosialin and thrombomodulin constitute a C-type lectin family that are frequently up-regulated in tumor vessels [95, 101–106]. CLEC14A, CD93 and endosialin all bind to the secreted extracellular matrix associated protein multimerin-2 [107, 108]. The interaction between endothelial CD93 and MMRN2 regulates fibronectin deposition during glioma angiogenesis, and loss of endosialin, mainly expressed in pericytes, protects against development of fibrosis, suggesting that this protein family participates in regulating the extracellular matrix [105, 109]. However, CD93-deficiency is associated with increased permeability, while endosialin expression in pericytes promotes intravasation of tumor cells and metastatic dissemination, indicating opposite roles in regulating vascular integrity [104, 110]. The specific transcriptional response of tumor endothelial cells is not only related to angiogenesis and vessel integrity, but may also affect endothelial activation and recruitment of leukocytes. Pro-angiogenic signaling leads to endothelial anergy, reduced response to pro-inflammatory signaling and decreased regulation of adhesion molecules and chemokines necessary for capture and trans-endothelial migration of leukocytes [111–114]. Up-regulation of FASL in tumor vessels further strengthens the endothelial barrier and contributes to immune suppression by inducing apoptosis of cytotoxic T-lymphocytes [115]. Similarly, expression of endothelin B in tumor vessels in ovarian cancer has been shown to decrease T cell homing [116]. Especially in brain tumors, the changes in endothelial gene expression induced by the tumor microenvironment can also be beneficial for therapy. The specific gene expression signature induced in tumor endothelial cells in WNT-medulloblastoma leads to disruption of the blood brain barrier and thereby renders the tumor sensitive to chemotherapy [117]. Proteins that are up-regulated in tumor vessels alter vascular function, and may constitute new targets for therapy as discussed further below.

## Growth factor and chemokine signaling in tumor angiogenesis

A large number of pro-angiogenic factors and their cognate receptors are known to promote vessel formation in tumors, including vascular endothelial growth factor (VEGF), fibroblast growth factor 2 (FGF-2), platelet derived growth factor (PDGF), angiopoietins, ephrins, apelin (APLN) and chemokines. These factors are often expressed simultaneously, effectively co-operating at different stages of tumor angiogenesis. The main functions and features of the most prominent pro-angiogenic factors are discussed briefly below.

### Vascular endothelial growth factors (VEGF)

The vascular endothelial growth factor family consists of five secreted proteins, VEGF (also referred to as VEGF-A), VEGF-B, VEGF-C, VEGF-D and placental growth factor (PlGF).

VEGF, originally identified as vascular permeability factor (VPF), is one of the most potent inducers of angiogenesis [118]. In cancer, VEGF is produced and secreted by tumor cells and surrounding stroma and is associated with tumor progression, increased vessel density, invasiveness, metastasis and tumor recurrence [119].

VEGF is up-regulated during hypoxia and orchestrate blood vessel formation mainly via activation of VEGF receptor-2 (VEGFR-2) expressed by endothelial cells [120]. VEGFR-2 activation initiates several signaling pathways leading to specific endothelial responses such as cell survival, proliferation, migration, invasion, vascular permeability and vascular inflammation [121]. A tight coordination of these cellular processes is crucial for a successful establishment of new vessels. During tumor angiogenesis, VEGF secreted by tumor cells induces endothelial cell proliferation and survival primarily via the ERK and PI3K/Akt pathways [122, 123]. Endothelial cell migration downstream of VEGFR2 is induced via multiple signaling pathways, often involving PI3K stimulation and activation of Rho GTPases [124]. On the other hand, VEGF-mediated cell invasion is promoted by the expression of MT (membrane type)-MMP (matrix metalloproteinase), MMP-2, MMP-9 and urokinase plasminogen activator which degrade the basal membrane and extracellular matrix allowing migration of endothelial cells and the formation of capillary sprouts [123, 125].

Vascular permeability is crucial for normal tissue homeostasis and is considered a prerequisite for VEGF-induced angiogenesis. VEGF induces vascular permeability by several mechanisms, including junctional remodeling, induction of fenestrae, and vesiculo-vascular organelles (VVOs) [126]. In pathological conditions such as cancer, dysregulation of these mechanisms leads to vascular hyper-permeability that in turn may exert direct effects on the tumor microenvironment including increased interstitial pressure and impaired therapeutic delivery [127]. Moreover, the leaky vasculature may facilitate the escape of tumor cells into the bloodstream promoting the establishment of distant metastases [128].

Vascular permeability is tightly related to vascular inflammation. Although VEGF is not an inflammatory cytokine, VEGF can induce the activation of the transcription factor NFAT in endothelial cells via PLCγ/calcineurin, promoting an inflammatory gene expression pattern similar to that of IL-1β [129]. In addition, VEGF-mediated activation of NF‐κB downstream of Akt can induce an inflammatory‐type response, promoting the attraction of leukocytes that can contribute to the angiogenic process. [123].

PlGF is a member of the VEGF family; however, its role in modulating tumor angiogenesis has been a subject of controversy. PlGF has been reported to enhance pathological angiogenesis by initiating a cross-talk between VEGFR-1 and VEGFR-2 [130], while others have demonstrated anti-angiogenic properties of PlGF [131]. Similarly, there have been contradictory results regarding the efficiency of anti-PlGF therapy in inhibiting angiogenesis and halting tumor growth in preclinical tumor models [132, 133].

### Fibroblast growth factor-2 (FGF2)

The mammalian fibroblast growth factor (FGF) family comprises 22 molecules, 18 of which interact with high affinity to tyrosine kinase receptors FGFR1, FGFR2, FGFR3 and FGFR4 [134]. FGFs are secreted glycoproteins that are sequestered in the extracellular matrix. To signal, FGFs are released from the extracellular matrix by heparinases, proteases or specific FGF binding proteins, and the liberated FGFs subsequently bind to cell surface heparan sulphate proteoglycans (HPSGs) stabilizing the FGF-FGFR interaction [135].

FGFs that signal through FGFR regulate a broad spectrum of biological functions and can involve both tumor cells and the surrounding stroma. These effects include cellular proliferation, resistance to cell death, increased motility and invasiveness, enhanced metastasis as well as increased angiogenesis [134]. FGF-2, also known as basic FGF (bFGF), is the most characterized pro-angiogenic mediator in physiological conditions as well as during tumor progression [136, 137]. FGF-2 exerts its effects on endothelial cells via a paracrine signaling after being released by tumor and stromal cells or mobilized from extracellular matrix. It has been described that FGF-2 can promote angiogenesis acting together with VEGF, by inducing the secretion of MMPs, plasminogen activator and collagenase responsible for the degradation and organization of the extracellular matrix [134]. In addition, a recent study has identified FGF signaling as a key regulator of blood and lymphatic vascular development by modulating endothelial metabolism driven by MYC-dependent glycolysis, which is crucial for endothelial cell sprouting, migration and proliferation [138]. In tumors, FGF expression has been associated with resistance to anti-angiogenic therapy. Indeed, activation of the proangiogenic FGF signaling pathway has been proposed to be a mechanism that the tumor cells use to escape from VEGF-targeted therapies. A recent study performed in a murine breast cancer model shows that FGF receptor inhibition leads to decreased vessel density and restored tumor sensitivity to anti-VEGF therapy [139].

### The platelet derived growth factor (PDGF) family

The PDGF family comprise four heparin-binding polypeptide growth factors denoted A, B, C, and D. PDGF is secreted by activated platelets, endothelial, epithelial, glial cells as well as inflammatory cells and it targets a broad spectrum of cell type including, fibroblasts, pericytes, smooth muscle cells, glial cells or mesangial cells [140]. PDGF signals through two cell-surface tyrosine kinase receptors, PDGFRα and PDGFRβ, and regulates many biological functions including angiogenesis, by promoting vessel maturation and recruitment of pericytes and by inducing upregulation of VEGF [141]. All members of the PDGF family display potent angiogenic activity in vivo, however, the PDGF-B/PDGFRβ axis is the most characterized. The importance of PDGF in vessel function was demonstrated by lethality of mice lacking components of the PDGF-B/PDGFRβ pathway, displaying vessel leakage and micro-hemorrhage [142].

PDGF and PDGFR are involved in cancer development and progression through autocrine stimulation of tumor cell growth and paracrine stimulation on stromal cells inducing tumor-associated angiogenesis. In an experimental model of glioma, PDGF-B enhanced angiogenesis by stimulating VEGF expression in tumor-associated endothelial cells and by recruiting pericytes in newly-formed vessels [143].

### Angiopoietins

Angiopoietins (ANGPTs) are growth factors that regulate development, maintenance and remodeling of the blood vessels, and they play a key role in controlling tumor growth and angiogenesis. The human angiopoietin family comprises the ligands ANGPT-1, ANGPT-2, and ANGPT-4 [144, 145]. Angiopoietins signaling is mediated by endothelial receptor tyrosine kinases TIE-1 and TIE-2, TIE-2 being the best characterized [146].

ANGPT-1 and ANGPT-2 both bind to TIE-2, but elicit very different responses. ANGPT-1 promotes vessel maturation and stabilization of the newly-formed vessels through the Akt/survivin pathway. In contrast, ANGPT-2 has been shown to induce vessel destabilization, pericytes detachment, vessel sprouting and angiogenesis [147]. Increased ANGPT-2 expression has been observed in activated endothelial cells during inflammation and in tumor-associated vessels of several human cancers in response to hypoxia and VEGF [148]. Moreover, ANGPT-2 has been identified as an autocrine regulator of endothelial cell inflammatory response by sensitizing endothelial cells towards TNF and inducing upregulation of adhesion molecules [149].

Upregulation of ANGPT-2 in glioblastoma has been associated with reduced efficacy of anti-VEGF treatment and increased therapy resistance [150]. Preclinical studies have demonstrated beneficial effects on inhibiting tumor progression by dual inhibition of ANGPT-2/VEGFR2. Indeed, simultaneous ANGPT-2 and VEGFR2 inhibition impairs tumor growth, prolong vessel normalization and blocks macrophage recruitment improving survival of glioma bearing mice [151, 152]. Co-targeting of ANGPT-2/VEGFR2 is also effective in other murine tumor models, including early breast cancer, colorectal and renal cancer [153].

### Eph/ephrin signalling

The Eph proteins belong to the superfamily of receptor tyrosine kinases and include 14 human type 1 transmembrane protein members. The Eph proteins are divided in two subgroups, EphA and EphB based on their sequence homologies and the ability to bind their ligands, the ephrins. The EphA subgroup includes nine members (EphA1-A8 and A10) and the EphB subgroup five members (EphB1-B4, B6). Unlike other tyrosine kinases whose ligands are soluble proteins, the Ephs ligands are associated with the plasma membrane of expressing cells and are classified in two subclasses based on the type of membrane binding. The ephrins A include six members (A1–A6) and are attached to the membrane by a glycosylphosphatidyl-inositol (GPI) domain. The Ephrins B are single pass type 1 transmembrane proteins and this subclass includes three members (B1–B3) [154].

A unique features of Eph receptors and their membrane anchored ligands is their ability to mediate bi-directional signals (forward and reverse signal) between adjacent cells. The “forward signal” occur when Eph/ephrin signal transduce into receptor-binding cell and the “reverse signal” when the ligand-receptor interaction leads to transduction into the ligand-expressing cell, reviewed in [155].

Ephrins and Eph receptors are involved in several processes that occur during embryogenesis including vascular development, tissue-border formation, cell migration and axon guidance [156, 157]. However, an important role Eph/ephrins system has also been found in pathological conditions such as cancer [158, 159]. Many ephrins and Eph receptors are upregulated in human tumors such as breast, colon, liver, brain, prostate and melanoma and are often associated with tumor progression and metastasis [158, 159]. On the other hand, also downregulation of Eph receptors can lead to increased metastasis as shown for EphA1 in colorectal cancer, EphA7 in prostate carcinomas, and EphB6 in melanoma [160, 161].

Several studies directly associate Eph/ephrins system to tumor angiogenesis. Ogawa et al. [162] was one of the first to report tumor vasculature-specific expression of EphA2 and ephrinA1 in blood vessels of preclinical models of breast carcinoma and Kaposi’s sarcoma. Subsequently, it was found that blocking EphA receptor signalling using soluble EphA receptors decreases tumor vascular density, tumor volume and cell proliferation [163–165].

EphB4-ephrinB2 signalling was also associated with increased tumor angiogenesis and tumor progression [166] as well as with resistance to anti-angiogenic therapy [167]. Indeed, in this preclinical study of glioma, EphB4 overexpression was associated with alterations in vascular morphogenesis, pericyte coverage, cellular proliferation and apoptosis, inducing a vascular phenotype resistant to therapy. Furthermore, a recent study identified EphrinB2 as a regulator of perivascular invasion and proliferation of glioblastoma stem-like cells [168].

Importantly, a connection between ephrins and VEGF signalling has also been shown. In particular, it was found that ephrin-B2 is able to control VEGF signalling by inducing VEGFR2 and VEGFR3 internalization, thereby regulating angiogenesis and lymphangiogenesis in physiological conditions as well as during tumor progression [169, 170].

### Apelin/APLNR pathway

Apelin (APLN) is an endogenous peptide-ligand of the G protein-coupled receptor APJ (APLNR) [171]. The APLN/APLNR signaling pathway is involved in developmental angiogenesis, where the APLNR expression is predominantly restricted to the ECs of the developing vascular system and APLN regulates vascular patterning in the embryo [172–174]. APLN/APLNR signaling has key function in several physiological processes like cardiac function, angiogenesis, metabolism and body fluid homeostasis, and also in pathological conditions like heart failure, cancer, obesity and diabetes (reviewed in detail [175]).

The APLN/APLNR pathway is upregulated in malignant cells in many tumor types [174, 176, 177], as well as in tumor endothelial cells [178], and elevated Apelin levels are associated with disease progression and poor clinical outcome [176, 179–181]. Apelin expression in tumors is regulated by hypoxia [181] and is suggested to promote tumor growth in several ways. Apelin directly stimulates tumor cell proliferation [181–183], tumor cell migration and metastasis [184, 185]. Apelin also stimulates neoangiogenesis and microvascular proliferation within the tumor, leading to enhanced tumor growth [174, 176, 186, 187].

The clinical outcome of targeting APLN/APLNR pathway for cancer therapy depends on the tumor type. In models of lung and breast cancer, targeting Apelin prevented resistance associated with anti-angiogenic therapy by reducing tumor growth, metastasis and improving vessel function [188]. In models of glioma, targeting Apelin promoted invasiveness of tumor cells positive for APLNR. However, combined targeting of VEGFR2 and Apelin improved survival of glioma bearing mice [189]. In another glioma study targeting APLNR with a competitive antagonist reduced tumor growth in mice [190]. In a renal cell carcinoma study, APLNR expression in a subset of patients was found to be negatively correlated with tumor PD-L1 expression [177]. This also indicates a role of APLN/APLNR signaling in the regulation of immunological processes, which needs to be further investigated.

### Chemokines

Chemokines are a large family of small secreted proteins with conserved cysteine residues that act through binding G-protein linked chemokine receptors with seven transmembrane structures. Depending on the number of amino acids separating the cysteine residues that make up the disulfide bonds that are required for structural integrity, chemokines are classified into CC, CXC, XC and CX3C subclasses [191]. The CXC chemokines are further divided into ELR + or ELR− groups depending on the presence or absence of a Glu-Leu-Arg motif preceding the first cysteine residue in the N-terminus, which is essential to regulate chemotaxis across endothelium. Chemokines mediate specific homing of progenitor cells and leukocytes expressing their cognate receptors. In cancer, chemokines contribute to tumor angiogenesis either directly through binding chemokine receptors expressed on endothelial cells, or indirectly through recruitment of inflammatory cells and progenitors.

ELR + CXC chemokines, including CXCL1, CXCL2, CXCL3, CXCL5, CXCL6, CXCL7 and CXCL8 enhance angiogenesis through binding to their common receptor CXCR2. CXCR2 can be expressed in microvascular endothelial cells [192], and in tumor vessels in several types of human cancer [193, 194]. Inhibition of CXCR2 decreased tumor growth and angiogenesis in a genetic murine model of pancreatic ductal adenocarcinoma [195]. In human ovarian carcinoma cells, CXCR2 activation induced angiogenesis through enhanced expression of VEGF and knockdown reduced tumorigenesis in nude mice [196]. Expression of ELR + chemokines may also induce angiogenesis indirectly, since CXCR2 can be expressed on neutrophils and is involved in leukocyte arrest prior to transendothelial migration [197]. Among the CXC ELR + chemokines, especially CXCL8 has been found to be important for tumor angiogenesis in several tumor types [198, 199]. It can support endothelial survival and induce release of pro-angiogenic factors such as VEGF, MMP-2 and MMP-9 [200–203]. CXCL8 is a strong neutrophil attractant, and induces neutrophil respiratory burst upon recruitment [204].

CXCL12/SDF1 binds to CXCR4 and is the only CXC ELR- chemokine that is directly pro-angiogenic and chemotactic, while other chemokines in this group, including CXCL4, CXCL9 CXCL10, CXCL11 and CXCL14 have angiostatic effects [205]. CXCR4 is enriched in tip cells and highly expressed in tumor vessels [11, 206]. Hypoxia-induced stabilization of HIF1a leads to up-regulation of CXCL12, which in turn mediates recruitment of CXCR4-expressing endothelial progenitor cells from the bone marrow, thereby contributing to vasculogenesis [207]. In addition, CXCL12/CXCR4 is involved in vessel co-option and trafficking of leukocytes to the tumor.

CCL2 is expressed in tumors and affects endothelial permeability and metastasis through interacting with CCR2 expressed on tumor endothelial cells [208]. Endothelial progenitor cells expressing CCR2 can be recruited from the circulation in response to tumor expression of CCL2, contributing to tumor angiogenesis [209]. The necessity of CCL2 for mobilization of endothelial progenitor cells was demonstrated in a genetic murine breast cancer model, exhibiting reduced numbers of these cells in the blood in Her2/neu CCL2-deficient mice [209]. Survival of Her2/neu mice was increased by treatment with CCXC872, a small molecule antagonist targeting CCR2.

### Other proangiogenic factors contribute to tumor angiogenesis

During tumor progression, expression of various matrix metalloproteases (MMPs) either by the tumor cells or by surrounding stromal cells, helps to remodel the ECM and release ECM- and membrane-bound growth factors promoting tumor progression, metastasis and tumor-associated angiogenesis. Transcription of MMPs can be induced by various signals including cytokines, growth factors, and mechanical stress. Secretion of MMP-2 and MMP-9 activate the latent form of transforming growth factor-beta (TGF-β), further promoting tumor invasion and angiogenesis [210]. TGF-β is an important regulator of neovascularization in tumor and it acts in a context-dependent manner by promoting angiogenesis via stimulation of pro-angiogenic factors like VEGF or inhibiting tumor angiogenesis by impairing endothelial cell proliferation and migration or by inducing apoptosis [211].

Tissue necrosis factor-α (TNF-α) is an inflammatory cytokine released by macrophages, mast cells and T-lymphocytes and it is also implicated in tumor progression, cell survival, differentiation, invasion, metastases as well as secretion of cytokines and pro-angiogenic factors. The effect of TNF on angiogenesis, however, is controversial. Indeed, it has been reported that depending on its temporal expression during the angiogenic process it can exert pro- or anti-angiogenic effects by regulating the expression of VEGFR2 [212].

Another pro-angiogenic factor expressed in tumors is pleiotrophin (PTN), a small heparin-binding cytokine that is abundant in the brain during embryonic development and is re-induced during pathological conditions [213]. PTN level is increased in several types of cancer including glioma, breast cancer, lung cancer, melanoma, neuroblastoma, pancreatic cancer, and prostate cancer, and may increase tumor growth either through direct effects on tumor cells or through stimulation of angiogenesis and remodeling of the tumor microenvironment [214, 215].

High levels of PTN correlates with poor survival of patients with astrocytomas and is associated with vascular abnormalities. Studies in murine glioma models have provided evidence that PTN can enhance tumor growth through stimulation of the tumor vasculature [216].

Many other factors potentially regulating angiogenesis in tumors have been identified, but have not yet been fully explored. Neurite outgrowth inhibitor or Nogo belongs to the reticulon 4 (RTN4) protein families, which consists of three major splicing isoforms (NogoA, Nogo-B, and Nogo-C) with distinct expression patterns that binds to NgR receptors [217, 218]. An essential role of Nogo-B in regulating vascular remodeling was reported in Nogo-A/B-deficient mice [219]. Mice that are deficient for Nogo-A/B exhibit reduced arteriogenesis and angiogenesis in vivo due to impaired macrophage infiltration [219, 220]. More recently it has been reported that the expression level of Nogo-B is upregulated in hepatocellular carcinoma and Nogo-B deficiency suppressed the tumor growth and metastasis [221]. The expression level of Nogo-B correlated with tumor vessel density in hepatocellular carcinoma and anti-Nogo-B antibody inhibited tumor growth in vivo via suppressing tumor angiogenesis [222].

## Hypoxia or genetic alterations leading to stabilization of HIF induce tumor angiogenesis

Hyper-proliferation of tumor cells results in increased oxygen consumption, and when the tumor mass surpass the blood supply the tumor becomes hypoxic. Hypoxia induces production of pro-angiogenic factors leading to enhanced, rapid and chaotic blood vessel formation. Cellular adaptation to hypoxia is primarily mediated by a family of transcriptional regulators, hypoxia-inducible factors (HIFs). HIFs are heterodimers consisting of an oxygen-dependent α-subunit (HIF-α) and an oxygen-independent β-subunit (HIF-β). HIF-α has three isoforms, HIF-1α, HIF-2α, and HIF-3α. HIF-1α being the major responsible for activating transcriptional responses under hypoxia [223]. Hypoxia-induced stabilization of HIF-1α, promote the upregulation of several pro-angiogenic genes including VEGF, FGF and PDGF [224].

Genetic alterations in the oxygen-signaling pathway can influence the activation of HIF under normoxic condition. The von Hippel-Lindau (VHL) protein plays a central role in the oxygen-sensing pathway promoting HIFα proteosome-mediated degradation during normoxia. Mutations in this gene, resulting in the stabilization of HIF-1α and activation of the target pro-angiogenic genes is found in many tumors and it is associated with tumor progression and poor patient outcome [225].

## Contributions of immune cells to tumor angiogenesis

The tumor microenvironment is comprised of a broad array of stromal cells, endothelial cells, immune and inflammatory cells. The malignant cells and cells within the tumor microenvironment continuously interact with each other to develop a dynamic and tumor-promoting milieu [226]. Notably, there is tight and mutual interplay between the immune and endothelial cells. Immune cells depend on the expression of adhesion molecules on endothelial cells for extravasation into tumor tissue, where they can exhibit their anti-tumor properties. On the other hand, immune cells are a source for several soluble factors that influence angiogenesis, endothelial cell behavior and subsequent tumor progression. The impact of different immune subsets on angiogenesis and endothelial cell remodeling is well studied [227–229]. The contribution of the most prominent immune cell types (macrophages, myeloid derived suppressor cells, neutrophils and lymphocytes) to tumor angiogenesis and endothelial cell remodeling are discussed below.

### Macrophages

Macrophages are specialized phagocytes that clear invading microbes and cell debris, present antigens to the adaptive immune system and release various immunomodulatory cytokines. They are very plastic cells, able to exist in a range of different phenotypes based on stimuli in the tissue microenvironment [230]. The two extremes of this range are the pro-inflammatory M1 phenotype, associated with active microbial killing, and the M2 phenotype, associated with immune suppression, tissue remodeling and angiogenesis [231]. Tumor-associated macrophages (TAMs) can have different phenotypes depending on the tumor microenvironment, but generally closely resemble M2 macrophages [232].

TAMs are known to modulate and support angiogenesis. Depletion of TAMs results in the inhibition of tumor angiogenesis, whereas reconstitution of TAMs promotes angiogenesis in murine cancer models [233, 234]. Hypoxia in the tumor microenvironment simulates metabolic adaptation of TAMs and pro-angiogenic characteristics. Primarily, TAMs promote angiogenesis by producing multiple proangiogenic factors facilitating the proliferation of endothelial cells, induction of sprouting, tube formation, and maturation of new blood vessels. These factors include VEGFA, VEGFC, VEGFD, EGF, FGF2, chemokines (CXCL8, CXCL12, TNFα and MCP-1), semaphorin 4D, adrenomedullin, and thymidine phosphorylase [231, 235–237, 237]. TAMs release a number of angiogenesis-modulating molecules that include enzymes (COX-2, iNOS) [238], matrix metalloproteinases (MMPs-1, 2, 3, 9, and 12) [239], cathepsin proteases [240] and plasmin, urokinase plasminogen activator [241]. They act in synergy and trigger degradation of the basement membrane and extracellular matrix components, destabilizing the vasculature and promoting migration and proliferation of endothelial cells. TAMs can also promote angiogenesis by inhibiting the expression of angiogenesis inhibitors, such as vasohibin-2 [242]. TAMs expressing Tie2 (TEMs) have been identified to be closely associated with the blood vessel and transmit angiogenic signals at least partially by the expression of FGF-2 [80]. TEMs support vessel stability by antagonizing the effect of vascular disrupting agents and promoting tumor growth [243].

### Myeloid-derived suppressor cells (MDSCs)

MDSCs are a heterogeneous population of immature myeloid cells that expand and accumulate under pathological conditions such as infection, trauma, autoimmune diseases and cancer. MDSCs are broadly classified in two sub-populations, the monocytic MDSC (M-MDSC) and granulocytic MDSC (G-MDSC), which exist both in humans and mice [244] [245]. However, there are no clear set of markers to differentiate G-MDSCs and neutrophils, and there has therefore been a debate and confusion in the field concerning the identity and relationship between these two cell types [246]. MDSC recruitment to the tumor can be induced by many different factors e.g. CSF3, IL-1β, and IL-6, and subsequently lead to activation of STAT3, rendering them potent as proangiogenic and immunosuppressive cells [247].

The capability MDSC regulating tumor angiogenesis is similar to M2-like TAMs. MDSCs promote and sustain tumor angiogenesis primarily by secretion of MMPs. In particular, MMP-9 is known to boost angiogenesis and stimulate tumor neovasculature by increasing the bioavailability of VEGF [248]. This initiates a feedforward loop as VEGF can further trigger MDSC recruitment [249]. MDSC accumulation in the tumor correlates with intra-tumoral VEGF concentration during disease progression [250]. In the presence of VEGF, MDSCs can create a pro-angiogenic milieu within the tumors by secreting angiogenic factors including CCL2, CXCL8, CXCL2, IL-1β, ANGPT1, ANGPT2, and GM-CSF [251, 252]. These chemokines can further promote MDSCs accumulation in the tumor creating a vicious circle. They also express Bv8, also known as prokineticin 2, which plays an important role in MDSC mediated angiogenesis [253]. Accumulation of MDSCs in the tumor microenvironment induces resistance to anti-angiogenic therapy [254, 255], while MDSC ablation has synergistic effects with anti-VEGF/VEGFR treatment [249, 256].

### Neutrophils

Neutrophils are the most abundant leukocyte population, providing the first line of defense against invading pathogens. They are a rich source of soluble factors such as ROS, peptides, cytokines and enzymes that exert antimicrobial activities [257]. Neutrophils are one of the main sources of VEGF and are known to play an important role during physiological angiogenesis, for example in endometrial angiogenesis during the menstrual cycle [258, 259]. Other studies have demonstrated that depletion of neutrophils affects neo-vascularization in animal models of angiogenesis [260, 261].

Conclusive evidence of neutrophils involvement in tumor angiogenesis came from studies in the RIP1-Tag2 multi-step pancreatic carcinogenesis mouse model. Neutrophil depletion using anti-GR1 antibodies reduced the number of dysplastic islets that were undergoing angiogenesis [262]. In addition, two subtypes of neutrophils have been reported at least in murine tumor models: TGFβ-independent type 1 (N1) with antimicrobial functions, and TGFβ-dependent tumor-associated neutrophils (N2, TANs) possessing pro-tumor and proangiogenic functions [263, 264]. Neutrophil survival and proliferation in tumors depend on CSF3-CSF3R mediated activation of STAT3 signaling. STAT3 activation in neutrophils triggers the angiogenic switch through secretion of VEGF, IL-8, TNF-α, MMP9, FGF2, ANGPT-1 and HGF in mice [265–267]. CSF3 is also known to stimulate neutrophils to secrete Bv8 and induce myeloid cell mobilization in tumors and promote myeloid-dependent angiogenesis [253]. MMP9-producing TANs contribute to the initiation of angiogenic switch and acceleration of tumorigenesis [262]. TANs usually lack expression of tissue inhibitors of metalloproteinases (TIMP1), rendering them more angiogenic than cells that are capable of producing TIMP1/MMP9 complexes [268].

### Lymphocytes

There are three major types of lymphocytes, namely T cells and B cells, which constitute the adaptive immune system and NK cells, which are part of the innate immune system. The contribution of lymphocytes towards tumor angiogenesis is not as well understood, as that of myeloid cell types.

A subset of NK cells (CD56^bright^CD16^−^KIR^+^, dNK cells), characterized by poor cytotoxicity and pro-angiogenic capacity have been identified in the decidua during pregnancy. They secrete VEGF, placental growth factor (PlGF), IFNγ, IL10 and CXCL8 that are critical for spiral artery formation and decidual vascularization [269, 270]. TGFβ promotes dNK cell polarization and can induce VEGF and PlGF secretion from healthy donor NK cells [271, 272]. In the presence of TGFβ, NK cells convert to type 1 innate lymphoid cells, leading to evasion of immune response and an inability to control tumor growth and metastasis [273].

The ability of B cells to modulate tumor angiogenesis depends on activation of STAT3. Transfer of B cells expressing STAT3 to Rag1^−/−^ mice leads to enhanced tumor growth accompanied with increased angiogenesis. This is a result of an interaction between STAT3-activated B cells and endothelial cells through production of VEGF [274]. B cells also contribute to tumor angiogenesis via antibody-mediated activation of Fcγ receptors on TAMs, inducing secretion of IL-1. This leads to recruitment of myofibroblasts and promotion of tumor angiogenesis [275].

T cells promote angiogenesis by secretion of pro-angiogenic factors FGF-2 and heparin-binding epidermal-like growth factor (HB-EGF) [276]. However, the most prominent T cell derived factors, such as TNF, TGFβ, and interferons (IFNs), have anti-angiogenic functions [277–279]. The antiangiogenic effects of IFNs are mediated by direct effects on endothelial cells and other cells in the tumor microenvironment. Treatment with IFN-α/β induced necrosis of endothelial cells within tumors and decreased tumor metastases to the liver and spleen [280]. In vitro, TNF and IFNs can block collagen synthesis and extracellular matrix formation and thus inhibit the formation of capillary-like structures [281, 282]. IFN-γ can inhibit neovascularization and induce apoptosis if endothelial cells in murine glioma models [277]. Type-I polarized T cells (Th1) secrete IFNγ and their presence in the tumor microenvironment usually correlates with good clinical outcome [283]. Interferon-induced CXC family chemokines inhibit endothelial cell proliferation, promote Th1 type T cell, NK and DC infiltration, thereby inhibiting tumor growth. CXCL9, CXCL10 and CXCL11 are interferon-inducible angiostatic chemokines that can directly inhibit angiogenesis by binding CXCR3 on endothelial cells [284–286].

## Anti-angiogenic therapy: successes and failures

The concept of targeting angiogenesis as a means to starve tumors was introduced by Judah Folkman and colleagues 48 years ago [1]. Since then, several antiangiogenic therapies, mainly targeting VEGF signaling pathway have been developed and approved for the treatment of a variety of tumors (Table 1). Despite promising results showed by pre-clinical studies, anti-VEGF monotherapy such as bevacizumab, sunitinib and aflibercept among others have only provided limited benefits in certain tumor types including advanced-stage renal cell carcinoma, hepatocellular carcinoma and colorectal carcinoma and have not shown efficacy in pancreatic adenocarcinoma, prostate cancer, breast cancer or melanoma [287]. Data obtained by the AVANT trial of adjuvant bevacizumab in colorectal cancer shows evidence of higher incidence of relapses and deaths in bevacizumab treated patients due to disease progression suggesting an increased tumor aggressiveness after anti-angiogenic therapy [288]. This is consistent with studies in experimental models of cancer, which correlate anti-angiogenic treatment with increased local tumor invasiveness and formation of distant metastasis [289–292]. In glioma, numerous clinical studies collectively show that anti-angiogenic treatment can prolong progression-free survival but fails to improve overall survival [293]. The limited success of anti-angiogenic therapy in glioma is likely at least in part due to an escape from therapy by invasive tumor cells co-opting the vasculature of the surrounding brain tissue. Several molecular mechanisms have been identified that may explain resistance and increased invasion after anti-angiogenic therapy in glioma, including mesenchymal transition of tumor cells, up-regulation of pro-angiogenic factors, activation of MET and up-regulation of MMPs [293–296]. Metastasis-promoting effects have mainly been obtained from experimental models and clear evidence from clinical studies is still lacking. The reasons underlying insufficient efficacy of vessel-targeting strategies have been extensively investigated, and include stroma and tumor cell mechanisms of resistance [287, 297].

**Table 1:** FDA approved anti-angiogenic drugs and their targets

Drug	Target molecule(s)	Tumor type	References
Monoclonal antibodies			
Bevacizumab	VEGF-A	Colorectal cancer, non-small cell lung cancer, cervical cancer, ovarian cancer, renal cell carcinoma, glioblastoma	[4, 335–339]
Ramucirumab	VEGFR-2	gastric or gastro-oesophageal junction cancers, colorectal cancer, hepatocellular carcinoma, non-small-cell lung carcinoma	[340–343]
Cetuximab	EGFR	Squamous cell carcinoma of the head and neck, colorectal cancer	[344, 345]
Panitumumab	EGFR	Colorectal cancer	[346]
Necitumumab	EGFR	Squamous non-small-cell lung cancer	[347]
Trastuzumab	HER2	HER2-positive breast cancer, HER2-positive advanced gastric or gastro-oesophageal junction cancer	[348, 349]
Pertuzumab	HER2	HER2-positive breast cancer	[350]
Tyrosine kinase inhibitors			
Sorafenib	VEGFR-1, VEGFR-2, VEGFR-3, PDGFR family, RAF	Hepatocellular carcinoma, renal cell carcinoma, thyroid cancer	[351–353]
Sunitinib	VEGFR-1, VEGFR-2, VEGFR-3, PDGFR family, Kit, FLT3, CSF-1R, RET	Gastrointestinal stroma tumor, pancreatic cancer, renal cell carcinoma	[354–356]
Imatinib	PDGFR, c-Kit, Abl	Gastrointestinal stroma tumor, myeloid leukemia, philadelphia chromosome-positive acute lymphoblastic leukemia	[357–359]
Pazopanib	VEGFR-1, VEGFR-2, VEGFR-3, PDGFR family, Kit, Itk, LcK, c-FMS	Renal cell carcinoma, soft tissue sarcoma	[360, 361]
Gefitinib	EGFR	Non-small cell lung cancer	[362]
Erlotinib	EGFR	Non-small cell lung cancer, pancreatic adenocarcinoma	[363, 364]
Vandetanib	VEGFR-2, FGFR family, RET, BRT, Tie-2,EPH, Src family	Medullary thyroid cancer	[365]
Regorafenib	VEGFR-2, VEGFR-3, PDGFR-β, RAF, RET, Kit	Colorectal cancer, Gastrointestinal stroma tumor, hepatocellular carcinoma	[366–368]
Neratinib	EGFR, HER-2	HER-2 positive breast cancer	[369]
Lapatinib	EGFR, HER-2	HER-2 positive breast cancer	[370]
Afatinib	EGFR, HER-2	Non-small cell lung cancer	[371]
Axitinib	VEGFR-1, VEGFR-2, VEGFR-3, PDGFR family, Kit	Renal cell carcinoma	[372]
Cabozantinib	VEGFR-2, c-Met	Hepatocellular carcinoma, medullary thyroid cancer, renal cell carcinoma	[373–375]
Lenvatinib	VEGFR-1, VEGFR-2, VEGFR-3, FGFRs, PDGFR-α, KIT, RET	Hepatocellular carcinoma, thyroid cancer	[376, 377]
Receptor fusion proteins			
Ziv-aflibercept (VEGF trap)	VEGF-A, VEGF-B, PlGF	Colorectal cancer	[378]
Immunomodulatory agents with anti-angiogenic effect			
Thalidomide	TNF-α, ILs, IFNs, VEGF, bFGF	Multiple myeloma	[379]
Lenalidomide	TNF-α, ILs, IFNs, VEGF, bFGF	Multiple myeloma	[380]
mTOR inhibitor with anti-angiogenic effect			
Everolimus	mTOR	Renal cell carcinoma, breast cancer, pancreatic cancer, gastrointestinal cancer, lung neuroendocrine tumor, subependymal giant cell astrocytoma	[381–385]

### Mechanisms of resistance to anti-angiogenic therapy

Resistance to anti-angiogenic therapy is an important issue that likely explains the variable response in different types of tumors and the limited overall survival benefits. Resistance can be classified into intrinsic resistance, observed from the outset of the therapy, and acquired resistance, observed after an initial positive response to therapy [297]. Several mechanisms have been proposed for anti-angiogenic therapy resistance, including direct effects of hypoxia such as induction of tumor invasion and metastasis, co-option of normal vessels in the surrounding tissue, vascular mimicry as well as the contribution of stromal cells including recruitment of TAMs, EPC and pro-angiogenic myeloid cells as well as the upregulation of alternative pro-angiogenic factors [297] [298].

As already mentioned, anti-angiogenic therapy can promote tumor invasion and metastasis in pre-clinical cancer models, which might be triggered by increased hypoxia due to vessel depletion. Indeed, the transcription of HIF-regulated genes controls different steps of tumor invasion and metastasis, including EMT, activation of MET signaling, recruitment of stromal cells, vascular mimicry and vessel co-option [299]. Vessel co-option is defined as a non-angiogenic process whereby tumor cells directly utilize the pre-existing vasculature of the non-malignant tissue as a supply of oxygen and nutrients, resulting in resistance to anti-angiogenic therapy [5]. The first evidence of vessel co-option as a mechanism of acquired resistance to anti-angiogenic therapy was demonstrated by a study in a mouse model of hepatocellular carcinoma investigating the response to sorafenib treatment [300]. In addition to vessel co-option, tumor cells can develop vascular mimicry as an alternative blood transportation system to counteract the lack of oxygen and nutrient upon anti-angiogenic therapy. Indeed, preclinical studies conducted in renal carcinoma model reported that the VEGFR2 inhibitor sunitinib increases vascular mimicry under hypoxia by transforming tumor cells into endothelial-like cells resulting in tumor resistance [301].

Recruitment of stromal cells, immune cells and progenitors is another potential mechanism for resistance to anti-angiogenic therapy. In particular, many studies have pointed out an important role of bone marrow derived cells (BMDCs) in this aspect. Recruitment of BMDCs in glioblastoma can cause resistance to vatalanib treatment and the depletion of BMDCs can potentiate the effects of this anti-angiogenic drug [302]. Release of proangiogenic factors and increased hypoxia in response to vascularization blockade can lead to recruitment of endothelial progenitor cells (EPC) from the bone marrow, which contribute to tumor vascularization and have been linked to development of resistance to anti-VEGF therapy [303]. Moreover, recruitment of pro-angiogenic myeloid cells is also considered to be a mechanism whereby tumors bypass the inhibitory effects of anti-angiogenics drugs. Tumors can recruit different populations of myeloid cells with pro-angiogenic properties which in turn can be used as an alternative source of pro-angiogenic chemokines and cytokines [304].

In addition, alternative pro-angiogenic signaling pathways including ANGPT-2, FGF-2, IL-8 can be induced by tumor cells in response to a pharmacological inhibition of the VEGF signaling pathway [297]. In recent years, progress has been made towards understanding the mechanism of action of anti-angiogenic drugs through evaluating the effects of anti-angiogenic inhibitors on tumor vessels in preclinical and clinical studies. An important aspect that have emerged is the broad spectrum of effects covered by the angiogenic inhibitors and the diversity in terms of therapeutic response [305].

## Mechanisms mediating the therapeutic effect of angiogenesis inhibitors

**Fig. 3:**
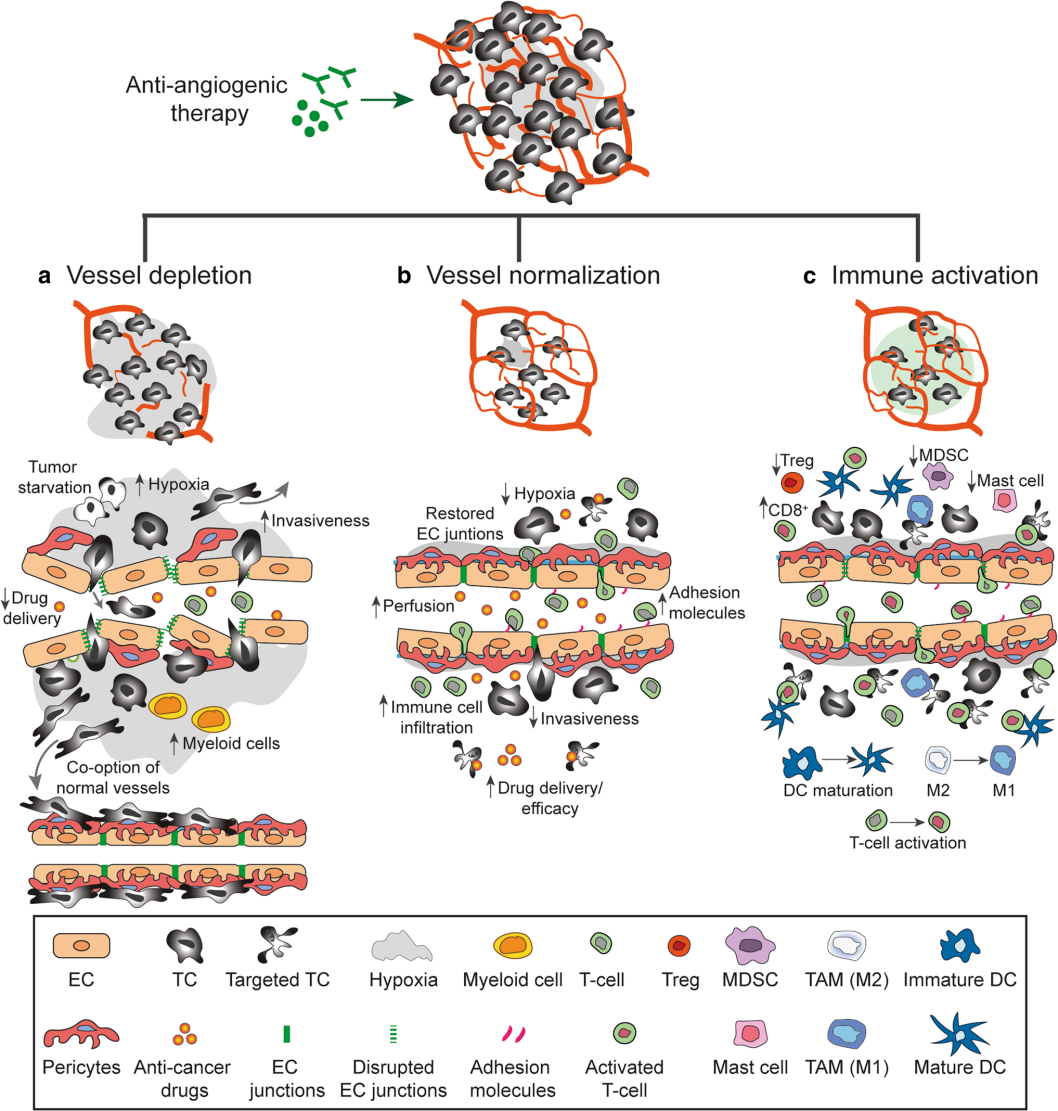
Effects of anti-angiogenic therapy. The mechanism of actions of angiogenesis inhibitors on tumor blood vessels can be classified into three categories: **a** vessel depletion, **b** vessel normalization, and **c** immune activation. **a** Vessel depletion result in tumor cell starvation and an increased tissue hypoxia. Enhanced hypoxia may promote the recruitment of pro-angiogenic myeloid cells and the mobilization of tumor cell from the hypoxic tissue to the normal tissue as well as co-option of normal vessels. In addition, the depletion of tumor vessels results in an inefficient delivery of anti-cancer drugs. **b** Normalization of tumor blood vessels achieved by restored endothelial cell junctions, increased pericytes coverage and re-established blood flow result in decrease tissue hypoxia and increased drugs delivery. In addition, vessel normalization promote the expression of endothelial adhesion molecule facilitating immune cell infiltration. **c** Immune activation, induced by anti-angiogenic drugs include dendritic cell (DC) maturation, activation and infiltration of T-cell as well as the polarization of tumor associated macrophages (TAM) towards an M1-like phenotype. In addition, a decrease in regulatory T-cells (Treg), myeloid derived suppressor cells (MDSCs) and mast cells have been observed in response to anti-angiogenic therapy

Although anti-angiogenic drugs were initially designed to block blood vessel formation, their ability to control tumor growth may be due to several different mechanism, which are not mutually exclusive. To improve vascular targeting, a thorough understanding of the cellular and molecular mechanisms that hinder tumor progression in response to anti-angiogenic therapy in specific tumors is necessary. The possible mechanism of actions of angiogenesis inhibitors on tumor blood vessels can be broadly classified into three categories: (a) vessel depletion, (b) vessel normalization, and (c) immune activation (Fig. 3).

### Vessel depletion

The development of anti-angiogenic drugs was initiated by the hypothesis that starving tumors by blocking angiogenesis would slow tumor progression and improve patient survival [1]. Early preclinical studies were promising and demonstrated a significant tumor growth delay and reduced metastasis. However, the effects of anti-angiogenic agents administrated as monotherapy in cancer patients during clinical trials often failed to show significant survival benefits. These observations suggest that anti-angiogenic therapy alone is insufficient to induce substantial tumor shrinkage in most cancer patients. Particular attention must be placed on the effects of tumor vessel depletion on the tumor microenvironment as well as the development of anti-angiogenic resistance. Indeed, as mentioned above, hypoxia induced by vessel depletion can activate several mechanisms used by tumor cells to counteract the lack of oxygen and nutrients such as increased tumor invasiveness and co-option of normal vessels resulting in ineffective anti-angiogenic therapy.

Several studies demonstrate that before reaching complete depletion of the vascular bed, anti-VEGF drugs induce an early and transient phase in which vessels assume normal shape and function [306, 307]. This vessel normalization window is characterized by a rescue of the balance between pro- and anti-angiogenic factors and it can promote increase tumor drug delivery and efficacy.

### Vessel normalization

Despite a high vascular density, tumors are usually hypoxic and nutrient-deprived since the tumor vessels are abnormal, leaky and malfunction. Such abnormal vasculature significantly compromises the efficacy of most anti-cancer therapies by limiting the delivery of drugs as well as promoting resistance to treatment.

The vessel normalization hypothesis, introduced by Rakesh Jain in 2001 [308] suggests that rather than depleting vessels, a sub-maximal doses of anti-angiogenic therapy can restore the normal function and structure of tumor vessels and improve drug delivery. This hypothesis could explain the increased progression-free survival observed in patients treated with anti-angiogenic drugs combined with chemotherapy as compared to treatment with chemotherapy alone [309]. Evidence supporting the idea that vessel normalization can improve cancer therapy has been obtained in mouse models. These studies show that improving tumor vessel perfusion and oxygenation ameliorates the efficacy of conventional therapies such as radiotherapy, chemotherapy and immunotherapy and reduces metastatic dissemination [309, 310].

Evidence that support the notion that vessel normalization occur in response to anti-angiogenic therapy has also been obtained from clinical studies. The functionality of the tumor vasculature in glioblastoma patients treated with anti-VEGF therapies has been evaluated by magnetic resonance imaging (MRI). MRI analysis of patients treated with cediranib revealed a decrease in vessel diameter, vascular permeability, and edema. More importantly, survival of patients with recurrent glioblastoma following cediranib-treatment was found to correlate with a vascular normalization index [311]. Improved perfusion occurred only in a subset of glioblastoma patients treated with cediranib, and was associated with improved patient overall survival [312]. These observations suggest that the degree of vessel normalization in terms of improved perfusion may be used as a tool to distinguish responders to anti-angiogenic therapy from non-responding patients [312, 313].

### Immune activation

Pro-angiogenic factors in tumors induce down-regulation of adhesion molecules on endothelial cells in the tumor vasculature and induce anergy to inflammatory signals such as TNFα and IL-1. Hereby, tumors with an angiogenic phenotype may escape the infiltration of cytotoxic leukocytes [111]. Using anti-angiogenic agents can potentially overcome the down-regulation of adhesion molecules and the unresponsiveness to inflammatory signals [314]. Consistent with this, normalization of tumor vasculature through anti-VEGF therapy in combination with adoptive T-cell transfer was found to increase tumor T-cell infiltration and improve survival in murine melanoma model [314].

Inhibition of VEGF signaling in the tumor microenvironment may be beneficial not only in terms of improving immune cell recruitment, but can also directly improve immune cell activation. Normalization of the tumor vascular network and decreased hypoxia can promote T cell infiltration and induce polarization of TAM to an M1-like phenotype [315]. Anti-angiogenic therapy can also reduce the prevalence of immunosuppressive cells. Decreases in Treg recruitment as well as MDSC has been reported after sunitinib treatment in tumor-bearing mice and in patients with metastatic renal carcinoma [316, 317]. In addition, inhibition of angiogenic signaling may improve T-cell priming and activation by improving dendritic cell (DC) maturation. Anti-anigogenic therapy using the VEGF-neutralizing antibody bevacizumab was found to increase the number and the maturation of DCs in patients with metastatic non-small cell lung carcinoma [318]. These observations indicate that immune activation is an additional mechanism that can contribute to response to anti-angiogenic therapy.

## Concluding remarks—arising opportunities for vascular targeting in cancer

Tumor vessels are often dysfunctional and anergic to inflammatory stimuli, leading to a hostile tumor microenvironment that fuel cancer progression and aggravate therapeutic approaches. Current vascular targeting strategies are based on inhibition of key angiogenic signaling pathways known to promote tumor angiogenesis. Although several anti-angiogenic drugs have been approved, intrinsic and acquired resistance to therapy limit their efficacy. An increased understanding of tumor vessel phenotype and mechanisms involved in treatment response and resistance to therapy is necessary to overcome the hurdles that prevent successful control of the angiogenic response in tumors. Alternatively, vascular targeting should instead be designed to target the tumor vessels in new ways that are conceptually different from inhibition of angiogenesis. This may involve altering the timing and dosing of already existing anti-angiogenic therapy in combination with other drugs, or development of novel therapeutics to either directly target the tumor vessels or optimize their function to fit the cancer therapy at hand.

### Differential gene expression in tumor vessels provides new tools for vascular targeting

The fact that tumor vessels differ molecularly from their normal counterparts can be used to develop treatment strategies that specifically target malignant cells and tumor vasculature. Therapeutic vaccination strategies to raise endogenous antibodies against antigens specifically expressed by tumor vasculature have shown efficacy in pre-clinical cancer models [319]. Prophylactic immunization of the alternatively spliced extra domain (ED)-B of fibronectin efficiently reduced growth of syngeneic subcutaneous tumors [320], and therapeutic vaccination against ED-A after tumor development reduced metastatic dissemination in the MMTV-PyMT model of metastatic mammary carcinoma [321]. Antibodies targeting tumor vessel markers have also been used. Blocking the tumor endothelial marker TEM8/anthrax toxin receptor 1 using antibodies raised against the extracellular domain inhibited angiogenesis, decreased growth of human tumor xenografts and increased the effect of anticancer drugs [322]. Conjugating TEM8-targeting antibodies with cytotoxic monomethyl auristatin E was successful in specifically directing the drug to the tumor microenvironment of orthotopic tumors and patient derived xenografts, significantly inducing regression or eradication of tumor growth in pre-clinical models [323]. Using an alternative strategy, targeting tumor endothelium and TEM8-positive malignant cells by employing TEM8-specific CAR T cells was effective in treating triple negative breast cancer (TNBC) patient derived xenograft (PDX) models and metastatic TNBC cell-line xenografts [324]. Peptides that specifically bind tumor endothelial cells have also been used to target either therapeutic antibodies or chemokines to the tumor microenvironment to improve efficacy and decrease toxicity [325, 326].

### Tailoring tumor vessels to optimize cancer therapy

Going beyond anti-angiogenesis and vascular normalization, strategies that can alter vessel phenotype to optimize specific types of cancer therapy are quickly emerging. It is already established that targeting VEGF/VEGFR signaling can enhance the efficacy of cancer immunotherapy by increasing expression of adhesion molecules and chemokines necessary for capture and transendothelial migration of T-cells [327, 328]. Several clinical trials have been initiated aimed at improving immunotherapy by combining checkpoint inhibitors with vascular targeting (http://clinicaltrials.gov) [327, 328]. To provide an even more efficient gateway for T-cells to enter the tumor microenvironment, tumor vessels can be induced to differentiate to high-endothelial venules (HEV). HEV have a distinct morphology, built up by cuboidal endothelial cells, and they express chemokine and adhesion molecules that mediate efficient recruitment of lymphocytes into the tissue [329]. Depletion of Tregs in a model of fibrosarcoma led to HEV neogenesis, enabling recruitment of T-cells into the tumor [330]. The presence of HEV within the tumor was a pre-requisite for tumor control after Treg depletion. Subsequently, it was found that activated CD8^+^ T cells induced HEV development within the tumor after Treg depletion [331]. Consistent with a role of activated T-cells in HEV neogenesis, combining anti-angiogenic therapy with anti-PD-L1 immunotherapy was sufficient to induce HEVs in several orthotopic and genetically engineered mouse models of cancer, stimulating tumor immunity [332]. Specific targeting of LIGHT/TNFSF14 to tumor vessels using vascular targeting peptides improved vessel functionality, activated endothelial cells and induced formation of HEV in murine glioblastoma, associated with enhanced accumulation of lymphocytes [333]. With respect to brain tumors, strategies that transiently open the blood brain barrier to enable delivery of drugs are of considerable interest [334]. The observation that paracrine signaling in WNT-medulloblastoma was associated with fenestrated tumor vessels that lack ABC transporters suggests that brain tumor vessels can indeed be modulated to allow a better penetration of drugs [117]. This exciting possibility has yet to be explored therapeutically. It is necessary to gain a deeper understanding of how tumor vessel function is altered in specific cancer types, and how vessel phenotype can be modulated. This may lead to new vascular targeting strategies aimed at tailoring vessel function to optimize drug response.

## References

[1] Folkman J (1971). Tumor angiogenesis: therapeutic implications. N Engl J Med.

[2] Folkman J (1971). Isolation of a tumor factor responsible for angiogenesis. J Exp Med.

[3] Cao Y (2011). Forty-year journey of angiogenesis translational research. Sci Transl Med.

[4] Hurwitz H (2004). Bevacizumab plus irinotecan, fluorouracil, and leucovorin for metastatic colorectal cancer. N Engl J Med.

[5] Kuczynski EA (2019). Vessel co-option in cancer. Nat Rev Clin Oncol.

[6] Hanahan D, Folkman J (1996). Patterns and emerging mechanisms of the angiogenic switch during tumorigenesis. Cell.

[7] Hanahan D (1985). Heritable formation of pancreatic beta-cell tumours in transgenic mice expressing recombinant insulin/simian virus 40 oncogenes. Nature.

[8] Nowak-Sliwinska P (2018). Consensus guidelines for the use and interpretation of angiogenesis assays. Angiogenesis.

[9] Jakobsson L, Bentley K, Gerhardt H (2009). VEGFRs and Notch: a dynamic collaboration in vascular patterning. Biochem Soc Trans.

[10] Tammela T (2008). Blocking VEGFR-3 suppresses angiogenic sprouting and vascular network formation. Nature.

[11] Strasser GA, Kaminker JS, Tessier-Lavigne M (2010). Microarray analysis of retinal endothelial tip cells identifies CXCR11 as a mediator of tip cell morphology and branching. Blood.

[12] Shawber CJ (2007). Notch alters VEGF responsiveness in human and murine endothelial cells by direct regulation of VEGFR-3 expression. J Clin Invest.

[13] Jakobsson L (2010). Endothelial cells dynamically compete for the tip cell position during angiogenic sprouting. Nat Cell Biol.

[14] Hellstrom M (2007). Dll4 signalling through Notch1 regulates formation of tip cells during angiogenesis. Nature.

[15] Lobov IB (2007). Delta-like ligand 4 (Dll4) is induced by VEGF as a negative regulator of angiogenic sprouting. Proc Natl Acad Sci USA.

[16] Harrington LS (2008). Regulation of multiple angiogenic pathways by Dll4 and Notch in human umbilical vein endothelial cells. Microvasc Res.

[17] Funahashi Y (2010). Notch regulates the angiogenic response via induction of VEGFR-1. J Angiogenes Res.

[18] Gerhardt H (2003). VEGF guides angiogenic sprouting utilizing endothelial tip cell filopodia. J Cell Biol.

[19] Fantin A (2013). NRP1 acts cell autonomously in endothelium to promote tip cell function during sprouting angiogenesis. Blood.

[20] Segarra M (2012). Semaphorin 6A regulates angiogenesis by modulating VEGF signaling. Blood.

[21] Phng LK (2009). Nrarp coordinates endothelial Notch and Wnt signaling to control vessel density in angiogenesis. Dev Cell.

[22] Herwig L (2011). Distinct cellular mechanisms of blood vessel fusion in the zebrafish embryo. Curr Biol.

[23] Kochhan E (2013). Blood flow changes coincide with cellular rearrangements during blood vessel pruning in zebrafish embryos. PLoS One.

[24] Lenard A (2015). Endothelial cell self-fusion during vascular pruning. PLoS Biol.

[25] Lenard A (2013). In vivo analysis reveals a highly stereotypic morphogenetic pathway of vascular anastomosis. Dev Cell.

[26] Phng LK, Stanchi F, Gerhardt H (2013). Filopodia are dispensable for endothelial tip cell guidance. Development.

[27] Blum Y (2008). Complex cell rearrangements during intersegmental vessel sprouting and vessel fusion in the zebrafish embryo. Dev Biol.

[28] Betz C (2016). Cell behaviors and dynamics during angiogenesis. Development.

[29] Patan S (1992). Intussusceptive microvascular growth: a common alternative to capillary sprouting. Arch Histol Cytol.

[30] Burri PH, Tarek MR (1990). A novel mechanism of capillary growth in the rat pulmonary microcirculation. Anat Rec.

[31] Hellstrom M (1999). Role of PDGF-B and PDGFR-beta in recruitment of vascular smooth muscle cells and pericytes during embryonic blood vessel formation in the mouse. Development.

[32] Wilting J (1996). VEGF121 induces proliferation of vascular endothelial cells and expression of flk-1 without affecting lymphatic vessels of chorioallantoic membrane. Dev Biol.

[33] Crivellato E (2004). Recombinant human erythropoietin induces intussusceptive microvascular growth in vivo. Leukemia.

[34] Ribatti D (2005). Microvascular density, vascular endothelial growth factor immunoreactivity in tumor cells, vessel diameter and intussusceptive microvascular growth in primary melanoma. Oncol Rep.

[35] Nico B (2010). Intussusceptive microvascular growth in human glioma. Clin Exp Med.

[36] Patan S, Munn LL, Jain RK (1996). Intussusceptive microvascular growth in a human colon adenocarcinoma xenograft: a novel mechanism of tumor angiogenesis. Microvasc Res.

[37] Djonov V (2001). MMP-19: cellular localization of a novel metalloproteinase within normal breast tissue and mammary gland tumours. J Pathol.

[38] Risau W (1988). Vasculogenesis and angiogenesis in embryonic-stem-cell-derived embryoid bodies. Development.

[39] Risau W, Lemmon V (1988). Changes in the vascular extracellular matrix during embryonic vasculogenesis and angiogenesis. Dev Biol.

[40] Choi K (1998). Hemangioblast development and regulation. Biochem Cell Biol.

[41] Asahara T (1997). Isolation of putative progenitor endothelial cells for angiogenesis. Science.

[42] Bussolati B, Grange C, Camussi G (2011). Tumor exploits alternative strategies to achieve vascularization. FASEB J.

[43] Kioi M (2010). Inhibition of vasculogenesis, but not angiogenesis, prevents the recurrence of glioblastoma after irradiation in mice. J Clin Invest.

[44] Ahn JB (2010). Circulating endothelial progenitor cells (EPC) for tumor vasculogenesis in gastric cancer patients. Cancer Lett.

[45] Greenfield JP, Cobb WS, Lyden D (2010). Resisting arrest: a switch from angiogenesis to vasculogenesis in recurrent malignant gliomas. J Clin Invest.

[46] Chopra H (2018). Insights into endothelial progenitor cells: origin, classification, potentials, and prospects. Stem Cells Int.

[47] Schmidt A, Brixius K, Bloch W (2007). Endothelial precursor cell migration during vasculogenesis. Circ Res.

[48] Romagnani P (2005). CD14 + CD34low cells with stem cell phenotypic and functional features are the major source of circulating endothelial progenitors. Circ Res.

[49] Shin JW (2005). Isolation of endothelial progenitor cells from cord blood and induction of differentiation by ex vivo expansion. Yonsei Med J.

[50] Urbich C, Dimmeler S (2004). Endothelial progenitor cells: characterization and role in vascular biology. Circ Res.

[51] Reale A (2016). Functional and biological role of endothelial precursor cells in tumour progression: a new potential therapeutic target in haematological malignancies. Stem Cells Int.

[52] Asahara T (1999). VEGF contributes to postnatal neovascularization by mobilizing bone marrow-derived endothelial progenitor cells. EMBO J.

[53] Hattori K (2001). Vascular endothelial growth factor and angiopoietin-1 stimulate postnatal hematopoiesis by recruitment of vasculogenic and hematopoietic stem cells. J Exp Med.

[54] Kopp HG, Ramos CA, Rafii S (2006). Contribution of endothelial progenitors and proangiogenic hematopoietic cells to vascularization of tumor and ischemic tissue. Curr Opin Hematol.

[55] Chang EI (2007). Hypoxia, hormones, and endothelial progenitor cells in hemangioma. Lymphat Res Biol.

[56] Spring H (2005). Chemokines direct endothelial progenitors into tumor neovessels. Proc Natl Acad Sci USA.

[57] Nakamura N (2009). Adiponectin promotes migration activities of endothelial progenitor cells via Cdc42/Rac1. FEBS Lett.

[58] Maniotis AJ (1999). Vascular channel formation by human melanoma cells in vivo and in vitro: vasculogenic mimicry. Am J Pathol.

[59] Ricci-Vitiani L (2010). Tumour vascularization via endothelial differentiation of glioblastoma stem-like cells. Nature.

[60] Upile T (2011). Vascular mimicry in cultured head and neck tumour cell lines. Head Neck Oncol.

[61] Williamson SC (2016). Vasculogenic mimicry in small cell lung cancer. Nat Commun.

[62] Baeten CI (2009). Prognostic role of vasculogenic mimicry in colorectal cancer. Dis Colon Rectum.

[63] Sharma N (2002). Prostatic tumor cell plasticity involves cooperative interactions of distinct phenotypic subpopulations: role in vasculogenic mimicry. Prostate.

[64] Fausto N (2000). Vasculogenic mimicry in tumors. Fact or artifact?. Am J Pathol.

[65] Seftor RE (2012). Tumor cell vasculogenic mimicry: from controversy to therapeutic promise. Am J Pathol.

[66] Folberg R, Maniotis AJ (2004). Vasculogenic mimicry. APMIS.

[67] Angara K, Borin TF, Arbab AS (2017). Vascular mimicry: a novel neovascularization mechanism driving anti-angiogenic therapy (AAT) resistance in glioblastoma. Transl Oncol.

[68] Valyi-Nagy K (2012). Stem cell marker CD271 is expressed by vasculogenic mimicry-forming uveal melanoma cells in three-dimensional cultures. Mol Vis.

[69] Lin AY (2005). Distinguishing fibrovascular septa from vasculogenic mimicry patterns. Arch Pathol Lab Med.

[70] Comito G (2011). HIF-1alpha stabilization by mitochondrial ROS promotes Met-dependent invasive growth and vasculogenic mimicry in melanoma cells. Free Radic Biol Med.

[71] Angara K (2017). Vascular mimicry in glioblastoma following anti-angiogenic and anti-20-HETE therapies. Histol Histopathol.

[72] Li M (2010). Vasculogenic mimicry: a new prognostic sign of gastric adenocarcinoma. Pathol Oncol Res.

[73] Wang R (2010). Glioblastoma stem-like cells give rise to tumour endothelium. Nature.

[74] Mei X (2017). Glioblastoma stem cell differentiation into endothelial cells evidenced through live-cell imaging. Neuro Oncol.

[75] Bussolati B (2009). Endothelial cell differentiation of human breast tumour stem/progenitor cells. J Cell Mol Med.

[76] Alvero AB (2009). Stem-like ovarian cancer cells can serve as tumor vascular progenitors. Stem Cells.

[77] Zhao Y (2010). Endothelial cell transdifferentiation of human glioma stem progenitor cells in vitro. Brain Res Bull.

[78] Kulla A (2003). Analysis of the TP53 gene in laser-microdissected glioblastoma vasculature. Acta Neuropathol.

[79] Rodriguez FJ (2012). Neoplastic cells are a rare component in human glioblastoma microvasculature. Oncotarget.

[80] De Palma M (2005). Tie2 identifies a hematopoietic lineage of proangiogenic monocytes required for tumor vessel formation and a mesenchymal population of pericyte progenitors. Cancer Cell.

[81] Cheng L (2013). Glioblastoma stem cells generate vascular pericytes to support vessel function and tumor growth. Cell.

[82] Baluk P, Hashizume H, McDonald DM (2005). Cellular abnormalities of blood vessels as targets in cancer. Curr Opin Genet Dev.

[83] McDonald DM, Baluk P (2005). Imaging of angiogenesis in inflamed airways and tumors: newly formed blood vessels are not alike and may be wildly abnormal: Parker B. Francis lecture. Chest.

[84] Kimura H (1996). Fluctuations in red cell flux in tumor microvessels can lead to transient hypoxia and reoxygenation in tumor parenchyma. Cancer Res.

[85] Bennewith KL, Durand RE (2004). Quantifying transient hypoxia in human tumor xenografts by flow cytometry. Cancer Res.

[86] Hashizume H (2000). Openings between defective endothelial cells explain tumor vessel leakiness. Am J Pathol.

[87] Padera TP (2004). Pathology: cancer cells compress intratumour vessels. Nature.

[88] Abramsson A (2002). Analysis of mural cell recruitment to tumor vessels. Circulation.

[89] Morikawa S (2002). Abnormalities in pericytes on blood vessels and endothelial sprouts in tumors. Am J Pathol.

[90] Baluk P (2003). Abnormalities of basement membrane on blood vessels and endothelial sprouts in tumors. Am J Pathol.

[91] St Croix B (2000). Genes expressed in human tumor endothelium. Science.

[92] Zhang L (2003). Tumor-derived vascular endothelial growth factor up-regulates angiopoietin-2 in host endothelium and destabilizes host vasculature, supporting angiogenesis in ovarian cancer. Cancer Res.

[93] Carson-Walter EB (2001). Cell surface tumor endothelial markers are conserved in mice and humans. Cancer Res.

[94] Huang X (2010). Lymphoma endothelium preferentially expresses Tim-3 and facilitates the progression of lymphoma by mediating immune evasion. J Exp Med.

[95] Dieterich LC (2012). Transcriptional profiling of human glioblastoma vessels indicates a key role of VEGF-A and TGFbeta2 in vascular abnormalization. J Pathol.

[96] Roudnicky F (2013). Endocan is upregulated on tumor vessels in invasive bladder cancer where it mediates VEGF-A-induced angiogenesis. Cancer Res.

[97] Zhao Q (2018). Single-cell transcriptome analyses reveal endothelial cell heterogeneity in tumors and changes following antiangiogenic treatment. Cancer Res.

[98] Buckanovich RJ (2007). Tumor vascular proteins as biomarkers in ovarian cancer. J Clin Oncol.

[99] Zhang L (2018). IDH mutation status is associated with distinct vascular gene expression signatures in lower-grade gliomas. Neuro Oncol.

[100] Masiero M (2013). A core human primary tumor angiogenesis signature identifies the endothelial orphan receptor ELTD1 as a key regulator of angiogenesis. Cancer Cell.

[101] Hanly AM, Winter DC (2007). The role of thrombomodulin in malignancy. Semin Thromb Hemost.

[102] Maruno M (1994). Expression of thrombomodulin in astrocytomas of various malignancy and in gliotic and normal brains. J Neurooncol.

[103] Mura M (2012). Identification and angiogenic role of the novel tumor endothelial marker CLEC14A. Oncogene.

[104] Langenkamp E (2015). Elevated expression of the C-type lectin CD93 in the glioblastoma vasculature regulates cytoskeletal rearrangements that enhance vessel function and reduce host survival. Cancer Res.

[105] Lugano R (2018). CD93 promotes beta1 integrin activation and fibronectin fibrillogenesis during tumor angiogenesis. J Clin Invest.

[106] Christian S (2008). Endosialin (Tem1) is a marker of tumor-associated myofibroblasts and tumor vessel-associated mural cells. Am J Pathol.

[107] Khan KA (2017). Multimerin-2 is a ligand for group 14 family C-type lectins CLEC14A, CD93 and CD248 spanning the endothelial pericyte interface. Oncogene.

[108] Galvagni F (2017). Dissecting the CD93-Multimerin 2 interaction involved in cell adhesion and migration of the activated endothelium. Matrix Biol.

[109] Mogler C (2015). Hepatic stellate cell-expressed endosialin balances fibrogenesis and hepatocyte proliferation during liver damage. EMBO Mol Med.

[110] Viski C (2016). Endosialin-expressing pericytes promote metastatic dissemination. Cancer Res.

[111] Griffioen AW (1996). Tumor angiogenesis is accompanied by a decreased inflammatory response of tumor-associated endothelium. Blood.

[112] Griffioen AW (1996). Endothelial intercellular adhesion molecule-1 expression is suppressed in human malignancies: the role of angiogenic factors. Cancer Res.

[113] Dirkx AE (2003). Tumor angiogenesis modulates leukocyte-vessel wall interactions in vivo by reducing endothelial adhesion molecule expression. Cancer Res.

[114] Huang H (2015). VEGF suppresses T-lymphocyte infiltration in the tumor microenvironment through inhibition of NF-kappaB-induced endothelial activation. FASEB J.

[115] Motz GT (2014). Tumor endothelium FasL establishes a selective immune barrier promoting tolerance in tumors. Nat Med.

[116] Buckanovich RJ (2008). Endothelin B receptor mediates the endothelial barrier to T cell homing to tumors and disables immune therapy. Nat Med.

[117] Phoenix TN (2016). Medulloblastoma genotype dictates blood brain barrier phenotype. Cancer Cell.

[118] Ferrara N, Gerber HP, LeCouter J (2003). The biology of VEGF and its receptors. Nat Med.

[119] Apte RS, Chen DS, Ferrara N (2019). VEGF in signaling and disease: beyond discovery and development. Cell.

[120] Ferrara N (2004). Vascular endothelial growth factor: basic science and clinical progress. Endocr Rev.

[121] Claesson-Welsh L, Welsh M (2013). VEGFA and tumour angiogenesis. J Intern Med.

[122] Takahashi T (2001). A single autophosphorylation site on KDR/Flk-1 is essential for VEGF-A-dependent activation of PLC-gamma and DNA synthesis in vascular endothelial cells. EMBO J.

[123] Jiang BH, Liu LZ (2009). PI3 K/PTEN signaling in angiogenesis and tumorigenesis. Adv Cancer Res.

[124] Lamalice L, Le Boeuf F, Huot J (2007). Endothelial cell migration during angiogenesis. Circ Res.

[125] van Hinsbergh VW, Koolwijk P (2008). Endothelial sprouting and angiogenesis: matrix metalloproteinases in the lead. Cardiovasc Res.

[126] Weis SM, Cheresh DA (2005). Pathophysiological consequences of VEGF-induced vascular permeability. Nature.

[127] Azzi S, Hebda JK, Gavard J (2013). Vascular permeability and drug delivery in cancers. Front Oncol.

[128] Weis S (2004). Endothelial barrier disruption by VEGF-mediated Src activity potentiates tumor cell extravasation and metastasis. J Cell Biol.

[129] Hofer E, Schweighofer B (2007). Signal transduction induced in endothelial cells by growth factor receptors involved in angiogenesis. Thromb Haemost.

[130] Autiero M (2003). Role of PlGF in the intra- and intermolecular cross talk between the VEGF receptors Flt1 and Flk1. Nat Med.

[131] Schomber T (2007). Placental growth factor-1 attenuates vascular endothelial growth factor-A-dependent tumor angiogenesis during beta cell carcinogenesis. Cancer Res.

[132] Fischer C (2007). Anti-PlGF inhibits growth of VEGF(R)-inhibitor-resistant tumors without affecting healthy vessels. Cell.

[133] Bais C (2010). PlGF blockade does not inhibit angiogenesis during primary tumor growth. Cell.

[134] Turner N, Grose R (2010). Fibroblast growth factor signalling: from development to cancer. Nat Rev Cancer.

[135] Ornitz DM, Itoh N (2015). The fibroblast growth factor signaling pathway. Wiley Interdiscip Rev Dev Biol.

[136] Presta M (2005). Fibroblast growth factor/fibroblast growth factor receptor system in angiogenesis. Cytokine Growth Factor Rev.

[137] Compagni A (2000). Fibroblast growth factors are required for efficient tumor angiogenesis. Cancer Res.

[138] Yu P (2017). FGF-dependent metabolic control of vascular development. Nature.

[139] Incio J (2018). Obesity promotes resistance to anti-VEGF therapy in breast cancer by up-regulating IL-6 and potentially FGF-2. Sci Transl Med.

[140] Heldin CH, Westermark B (1999). Mechanism of action and in vivo role of platelet-derived growth factor. Physiol Rev.

[141] Franco M (2011). Pericytes promote endothelial cell survival through induction of autocrine VEGF-A signaling and Bcl-w expression. Blood.

[142] Betsholtz C (2004). Insight into the physiological functions of PDGF through genetic studies in mice. Cytokine Growth Factor Rev.

[143] Guo P (2003). Platelet-derived growth factor-B enhances glioma angiogenesis by stimulating vascular endothelial growth factor expression in tumor endothelia and by promoting pericyte recruitment. Am J Pathol.

[144] Davis S (1996). Isolation of angiopoietin-1, a ligand for the TIE2 receptor, by secretion-trap expression cloning. Cell.

[145] Maisonpierre PC (1997). Angiopoietin-2, a natural antagonist for Tie2 that disrupts in vivo angiogenesis. Science.

[146] Kiss EA, Saharinen P, Marmé D (2018). Anti-angiogenic targets: angiopoietin and angiopoietin-receptors. Tumor angiogenesis: a key target for cancer therapy.

[147] Reiss Y (2009). Switching of vascular phenotypes within a murine breast cancer model induced by angiopoietin-2. J Pathol.

[148] Shim WS, Ho IA, Wong PE (2007). Angiopoietin: a TIE(d) balance in tumor angiogenesis. Mol Cancer Res.

[149] Fiedler U (2006). Angiopoietin-2 sensitizes endothelial cells to TNF-alpha and has a crucial role in the induction of inflammation. Nat Med.

[150] Chae SS (2010). Angiopoietin-2 interferes with anti-VEGFR2-induced vessel normalization and survival benefit in mice bearing gliomas. Clin Cancer Res.

[151] Peterson TE (2016). Dual inhibition of Ang-2 and VEGF receptors normalizes tumor vasculature and prolongs survival in glioblastoma by altering macrophages. Proc Natl Acad Sci USA.

[152] Kloepper J (2016). Ang-2/VEGF bispecific antibody reprograms macrophages and resident microglia to anti-tumor phenotype and prolongs glioblastoma survival. Proc Natl Acad Sci USA.

[153] Wu FT (2016). Efficacy of cotargeting angiopoietin-2 and the VEGF pathway in the adjuvant postsurgical setting for early breast, colorectal, and renal cancers. Cancer Res.

[154] Lisle JE (2013). Eph receptors and their ligands: promising molecular biomarkers and therapeutic targets in prostate cancer. Biochim Biophys Acta.

[155] Kullander K, Klein R (2002). Mechanisms and functions of Eph and ephrin signalling. Nat Rev Mol Cell Biol.

[156] Holder N, Klein R (1999). Eph receptors and ephrins: effectors of morphogenesis. Development.

[157] Adams RH, Klein R (2000). Eph receptors and ephrin ligands. Essential mediators of vascular development. Trends Cardiovasc Med.

[158] Surawska H, Ma PC, Salgia R (2004). The role of ephrins and Eph receptors in cancer. Cytokine Growth Factor Rev.

[159] Dodelet VC, Pasquale EB (2000). Eph receptors and ephrin ligands: embryogenesis to tumorigenesis. Oncogene.

[160] Dong Y (2009). Downregulation of EphA1 in colorectal carcinomas correlates with invasion and metastasis. Mod Pathol.

[161] Hafner C (2003). Loss of EphB6 expression in metastatic melanoma. Int J Oncol.

[162] Ogawa K (2000). The ephrin-A1 ligand and its receptor, EphA2, are expressed during tumor neovascularization. Oncogene.

[163] Dobrzanski P (2004). Antiangiogenic and antitumor efficacy of EphA2 receptor antagonist. Cancer Res.

[164] Brantley DM (2002). Soluble Eph A receptors inhibit tumor angiogenesis and progression in vivo. Oncogene.

[165] Cheng N (2003). Inhibition of VEGF-dependent multistage carcinogenesis by soluble EphA receptors. Neoplasia.

[166] Noren NK (2004). Interplay between EphB4 on tumor cells and vascular ephrin-B2 regulates tumor growth. Proc Natl Acad Sci USA.

[167] Uhl C (2018). EphB4 mediates resistance to antiangiogenic therapy in experimental glioma. Angiogenesis.

[168] Krusche B (2016). EphrinB2 drives perivascular invasion and proliferation of glioblastoma stem-like cells. Elife.

[169] Wang Y (2010). Ephrin-B2 controls VEGF-induced angiogenesis and lymphangiogenesis. Nature.

[170] Sawamiphak S (2010). Ephrin-B2 regulates VEGFR2 function in developmental and tumour angiogenesis. Nature.

[171] Tatemoto K (1998). Isolation and characterization of a novel endogenous peptide ligand for the human APJ receptor. Biochem Biophys Res Commun.

[172] Devic E (1996). Expression of a new G protein-coupled receptor X-msr is associated with an endothelial lineage in Xenopus laevis. Mech Dev.

[173] Cox CM (2006). Apelin, the ligand for the endothelial G-protein-coupled receptor, APJ, is a potent angiogenic factor required for normal vascular development of the frog embryo. Dev Biol.

[174] Kalin RE (2007). Paracrine and autocrine mechanisms of apelin signaling govern embryonic and tumor angiogenesis. Dev Biol.

[175] Wysocka MB, Pietraszek-Gremplewicz K, Nowak D (2018). The role of apelin in cardiovascular diseases, obesity and cancer. Front Physiol.

[176] Berta J (2010). Apelin expression in human non-small cell lung cancer: role in angiogenesis and prognosis. J Thorac Oncol.

[177] Tolkach Y (2019). Apelin and apelin receptor expression in renal cell carcinoma. Br J Cancer.

[178] Seaman S (2007). Genes that distinguish physiological and pathological angiogenesis. Cancer Cell.

[179] Feng M (2016). Tumor apelin, not serum apelin, is associated with the clinical features and prognosis of gastric cancer. BMC Cancer.

[180] Lacquaniti A (2015). Apelin beyond kidney failure and hyponatremia: a useful biomarker for cancer disease progression evaluation. Clin Exp Med.

[181] Heo K (2012). Hypoxia-induced up-regulation of apelin is associated with a poor prognosis in oral squamous cell carcinoma patients. Oral Oncol.

[182] Hall C (2017). Inhibition of the apelin/apelin receptor axis decreases cholangiocarcinoma growth. Cancer Lett.

[183] Macaluso NJ (2011). Discovery of a competitive apelin receptor (APJ) antagonist. Chem Med Chem.

[184] Lv D (2016). PAK1-cofilin phosphorylation mediates human lung adenocarcinoma cells migration induced by apelin-13. Clin Exp Pharmacol Physiol.

[185] Berta J (2014). Apelin promotes lymphangiogenesis and lymph node metastasis. Oncotarget.

[186] Sorli SC (2007). Apelin is a potent activator of tumour neoangiogenesis. Oncogene.

[187] Sorli SC (2006). Therapeutic potential of interfering with apelin signalling. Drug Discov Today.

[188] Uribesalgo I (2019). Apelin inhibition prevents resistance and metastasis associated with anti-angiogenic therapy. EMBO Mol Med.

[189] Mastrella G (2019). Targeting APLN/APLNR improves antiangiogenic efficiency and blunts proinvasive side effects of VEGFA/VEGFR2 blockade in glioblastoma. Cancer Res.

[190] Harford-Wright E (2017). Pharmacological targeting of apelin impairs glioblastoma growth. Brain.

[191] Le Y (2004). Chemokines and chemokine receptors: their manifold roles in homeostasis and disease. Cell Mol Immunol.

[192] Heidemann J (2003). Angiogenic effects of interleukin 8 (CXCL8) in human intestinal microvascular endothelial cells are mediated by CXCR192. J Biol Chem.

[193] Keane MP (2004). Depletion of CXCR193 inhibits tumor growth and angiogenesis in a murine model of lung cancer. J Immunol.

[194] Kitadai Y (2000). Regulation of disease-progression genes in human gastric carcinoma cells by interleukin 8. Clin Cancer Res.

[195] Ijichi H (2011). Inhibiting Cxcr2 disrupts tumor-stromal interactions and improves survival in a mouse model of pancreatic ductal adenocarcinoma. J Clin Invest.

[196] Yang G (2010). CXCR196 promotes ovarian cancer growth through dysregulated cell cycle, diminished apoptosis, and enhanced angiogenesis. Clin Cancer Res.

[197] Smith ML, Olson TS, Ley K (2004). CXCR197- and E-selectin-induced neutrophil arrest during inflammation in vivo. J Exp Med.

[198] Smith DR (1994). Inhibition of interleukin 8 attenuates angiogenesis in bronchogenic carcinoma. J Exp Med.

[199] Ha H, Debnath B, Neamati N (2017). Role of the CXCL8-CXCR199/2 axis in cancer and inflammatory diseases. Theranostics.

[200] Martin D, Galisteo R, Gutkind JS (2009). CXCL8/IL8 stimulates vascular endothelial growth factor (VEGF) expression and the autocrine activation of VEGFR2 in endothelial cells by activating NFkappaB through the CBM (Carma3/Bcl10/Malt1) complex. J Biol Chem.

[201] Scapini P (2004). CXCL1/macrophage inflammatory protein-2-induced angiogenesis in vivo is mediated by neutrophil-derived vascular endothelial growth factor-A. J Immunol.

[202] Zhao X (2009). ELR-CXC chemokine receptor antagonism targets inflammatory responses at multiple levels. J Immunol.

[203] Li A (2005). Autocrine role of interleukin-8 in induction of endothelial cell proliferation, survival, migration and MMP-2 production and angiogenesis. Angiogenesis.

[204] Kobayashi Y (2008). The role of chemokines in neutrophil biology. Front Biosci.

[205] Sozzani S (2015). Chemokines as effector and target molecules in vascular biology. Cardiovasc Res.

[206] Xu J (2017). Vascular CXCR206 expression promotes vessel sprouting and sensitivity to sorafenib treatment in hepatocellular carcinoma. Clin Cancer Res.

[207] Ceradini DJ (2004). Progenitor cell trafficking is regulated by hypoxic gradients through HIF-1 induction of SDF-1. Nat Med.

[208] Wolf MJ (2012). Endothelial CCR208 signaling induced by colon carcinoma cells enables extravasation via the JAK2-Stat5 and p38MAPK pathway. Cancer Cell.

[209] Chen X (2016). CCL2/CCR209 regulates the tumor microenvironment in HER-2/neu-driven mammary carcinomas in mice. PLoS One.

[210] Yu Q, Stamenkovic I (2000). Cell surface-localized matrix metalloproteinase-9 proteolytically activates TGF-beta and promotes tumor invasion and angiogenesis. Genes Dev.

[211] Gupta MK, Qin RY (2003). Mechanism and its regulation of tumor-induced angiogenesis. World J Gastroenterol.

[212] Sainson RC (2008). TNF primes endothelial cells for angiogenic sprouting by inducing a tip cell phenotype. Blood.

[213] Muramatsu T (2002). Midkine and pleiotrophin: two related proteins involved in development, survival, inflammation and tumorigenesis. J Biochem.

[214] Lu KV (2005). Differential induction of glioblastoma migration and growth by two forms of pleiotrophin. J Biol Chem.

[215] Chen H (2009). Pleiotrophin produced by multiple myeloma induces transdifferentiation of monocytes into vascular endothelial cells: a novel mechanism of tumor-induced vasculogenesis. Blood.

[216] Zhang L (2015). Pleiotrophin promotes vascular abnormalization in gliomas and correlates with poor survival in patients with astrocytomas. Sci Signal.

[217] Cai Y (2005). Identification of a new RTN3 transcript, RTN3-A1, and its distribution in adult mouse brain. Brain Res Mol Brain Res.

[218] Yang J (2000). Assignment of the human reticulon 4 gene (RTN4) to chromosome 2p14– > 2p13 by radiation hybrid mapping. Cytogenet Cell Genet.

[219] Acevedo L (2004). A new role for Nogo as a regulator of vascular remodeling. Nat Med.

[220] Yu J (2009). Reticulon 4B (Nogo-B) is necessary for macrophage infiltration and tissue repair. Proc Natl Acad Sci USA.

[221] Zhu B (2017). Knockout of the Nogo-B gene attenuates tumor growth and metastasis in hepatocellular carcinoma. Neoplasia.

[222] Cai H (2018). Nogo-B promotes tumor angiogenesis and provides a potential therapeutic target in hepatocellular carcinoma. Mol Oncol.

[223] Lv X (2017). The role of hypoxia-inducible factors in tumor angiogenesis and cell metabolism. Genes Dis.

[224] Liao D, Johnson RS (2007). Hypoxia: a key regulator of angiogenesis in cancer. Cancer Metastasis Rev.

[225] Miller F (2005). Inactivation of VHL by tumorigenic mutations that disrupt dynamic coupling of the pVHL. Hypoxia-inducible transcription factor-1alpha complex. J Biol Chem.

[226] Hanahan D, Coussens LM (2012). Accessories to the crime: functions of cells recruited to the tumor microenvironment. Cancer Cell.

[227] de Visser KE, Coussens LM (2006). The inflammatory tumor microenvironment and its impact on cancer development. Contrib Microbiol.

[228] Benelli R (2006). Cytokines and chemokines as regulators of angiogenesis in health and disease. Curr Pharm Des.

[229] Albini A (2018). Contribution to tumor angiogenesis from innate immune cells within the tumor microenvironment: implications for immunotherapy. Front Immunol.

[230] Balkwill F, Charles KA, Mantovani A (2005). Smoldering and polarized inflammation in the initiation and promotion of malignant disease. Cancer Cell.

[231] Mantovani A, Sica A (2010). Macrophages, innate immunity and cancer: balance, tolerance, and diversity. Curr Opin Immunol.

[232] Biswas SK (2006). A distinct and unique transcriptional program expressed by tumor-associated macrophages (defective NF-kappaB and enhanced IRF-3/STAT1 activation). Blood.

[233] Lin EY (2006). Macrophages regulate the angiogenic switch in a mouse model of breast cancer. Cancer Res.

[234] Zhang W (2010). Depletion of tumor-associated macrophages enhances the effect of sorafenib in metastatic liver cancer models by antimetastatic and antiangiogenic effects. Clin Cancer Res.

[235] Spiric Z, Eri Z, Eric M (2015). Significance of vascular endothelial growth factor (VEGF)-C and VEGF-D in the progression of cutaneous melanoma. Int J Surg Pathol.

[236] Zhou H (2012). Semaphorin 4D cooperates with VEGF to promote angiogenesis and tumor progression. Angiogenesis.

[237] Cejudo-Martin P (2002). Hypoxia is an inducer of vasodilator agents in peritoneal macrophages of cirrhotic patients. Hepatology.

[238] Klimp AH (2001). Expression of cyclooxygenase-2 and inducible nitric oxide synthase in human ovarian tumors and tumor-associated macrophages. Cancer Res.

[239] Giraudo E, Inoue M, Hanahan D (2004). An amino-bisphosphonate targets MMP-9-expressing macrophages and angiogenesis to impair cervical carcinogenesis. J Clin Invest.

[240] Gocheva V (2010). IL-4 induces cathepsin protease activity in tumor-associated macrophages to promote cancer growth and invasion. Genes Dev.

[241] Zhang J (2011). Activation of urokinase plasminogen activator and its receptor axis is essential for macrophage infiltration in a prostate cancer mouse model. Neoplasia.

[242] Shen Z (2012). Vasohibin-1 and vasohibin-2 expression in gastric cancer cells and TAMs. Med Oncol.

[243] Welford AF (2011). TIE2-expressing macrophages limit the therapeutic efficacy of the vascular-disrupting agent combretastatin A4 phosphate in mice. J Clin Invest.

[244] Bronte V (2016). Recommendations for myeloid-derived suppressor cell nomenclature and characterization standards. Nat Commun.

[245] Parker KH, Beury DW, Ostrand-Rosenberg S (2015). Myeloid-derived suppressor cells: critical cells driving immune suppression in the tumor microenvironment. Adv Cancer Res.

[246] Coffelt SB, Wellenstein MD, de Visser KE (2016). Neutrophils in cancer: neutral no more. Nat Rev Cancer.

[247] Kumar V (2016). The nature of myeloid-derived suppressor cells in the tumor microenvironment. Trends Immunol.

[248] Jacob A, Prekeris R (2015). The regulation of MMP targeting to invadopodia during cancer metastasis. Front Cell Dev Biol.

[249] Horikawa N (2017). Expression of vascular endothelial growth factor in ovarian cancer inhibits tumor immunity through the accumulation of myeloid-derived suppressor cells. Clin Cancer Res.

[250] Karakhanova S (2015). Characterization of myeloid leukocytes and soluble mediators in pancreatic cancer: importance of myeloid-derived suppressor cells. Oncoimmunology.

[251] Chun E (2015). CCL2 promotes colorectal carcinogenesis by enhancing polymorphonuclear myeloid-derived suppressor cell population and function. Cell Rep.

[252] Obermajer N (2011). PGE(2)-induced CXCL12 production and CXCR252 expression controls the accumulation of human MDSCs in ovarian cancer environment. Cancer Res.

[253] Shojaei F (2007). Bv8 regulates myeloid-cell-dependent tumour angiogenesis. Nature.

[254] Piao Y (2012). Glioblastoma resistance to anti-VEGF therapy is associated with myeloid cell infiltration, stem cell accumulation, and a mesenchymal phenotype. Neuro Oncol.

[255] Hao Z, Sadek I (2016). Sunitinib: the antiangiogenic effects and beyond. Onco Targets Ther.

[256] van Hooren L (2016). Sunitinib enhances the antitumor responses of agonistic CD40-antibody by reducing MDSCs and synergistically improving endothelial activation and T-cell recruitment. Oncotarget.

[257] Tecchio C (2013). On the cytokines produced by human neutrophils in tumors. Semin Cancer Biol.

[258] Mueller MD (2000). Neutrophils infiltrating the endometrium express vascular endothelial growth factor: potential role in endometrial angiogenesis. Fertil Steril.

[259] Heryanto B, Girling JE, Rogers PA (2004). Intravascular neutrophils partially mediate the endometrial endothelial cell proliferative response to oestrogen in ovariectomised mice. Reproduction.

[260] Shaw JP (2003). Polymorphonuclear neutrophils promote rFGF-2-induced angiogenesis in vivo. J Surg Res.

[261] Benelli R (2002). Neutrophils as a key cellular target for angiostatin: implications for regulation of angiogenesis and inflammation. FASEB J.

[262] Nozawa H, Chiu C, Hanahan D (2006). Infiltrating neutrophils mediate the initial angiogenic switch in a mouse model of multistage carcinogenesis. Proc Natl Acad Sci USA.

[263] Shaul ME, Fridlender ZG (2017). Neutrophils as active regulators of the immune system in the tumor microenvironment. J Leukoc Biol.

[264] Fridlender ZG (2009). Polarization of tumor-associated neutrophil phenotype by TGF-beta: “N1” versus “N2” TAN. Cancer Cell.

[265] Grenier A (2002). Presence of a mobilizable intracellular pool of hepatocyte growth factor in human polymorphonuclear neutrophils. Blood.

[266] Dubravec DB (1990). Circulating human peripheral blood granulocytes synthesize and secrete tumor necrosis factor alpha. Proc Natl Acad Sci USA.

[267] Kujawski M (2008). Stat3 mediates myeloid cell-dependent tumor angiogenesis in mice. J Clin Invest.

[268] Ardi VC (2007). Human neutrophils uniquely release TIMP-free MMP-9 to provide a potent catalytic stimulator of angiogenesis. Proc Natl Acad Sci USA.

[269] Hanna J (2006). Decidual NK cells regulate key developmental processes at the human fetal-maternal interface. Nat Med.

[270] Blois SM, Klapp BF, Barrientos G (2011). Decidualization and angiogenesis in early pregnancy: unravelling the functions of DC and NK cells. J Reprod Immunol.

[271] Keskin DB (2007). TGFbeta promotes conversion of CD16 + peripheral blood NK cells into CD16- NK cells with similarities to decidual NK cells. Proc Natl Acad Sci USA.

[272] Bruno A (2013). The proangiogenic phenotype of natural killer cells in patients with non-small cell lung cancer. Neoplasia.

[273] Gao Y (2017). Tumor immunoevasion by the conversion of effector NK cells into type 1 innate lymphoid cells. Nat Immunol.

[274] Yang C (2013). B cells promote tumor progression via STAT3 regulated-angiogenesis. PLoS One.

[275] Andreu P (2010). FcRgamma activation regulates inflammation-associated squamous carcinogenesis. Cancer Cell.

[276] Blotnick S (1994). T lymphocytes synthesize and export heparin-binding epidermal growth factor-like growth factor and basic fibroblast growth factor, mitogens for vascular cells and fibroblasts: differential production and release by CD4 + and CD8 + T cells. Proc Natl Acad Sci USA.

[277] Fathallah-Shaykh HM (2000). Gene transfer of IFN-gamma into established brain tumors represses growth by antiangiogenesis. J Immunol.

[278] Friesel R, Komoriya A, Maciag T (1987). Inhibition of endothelial cell proliferation by gamma-interferon. J Cell Biol.

[279] Madri JA, Pratt BM, Tucker AM (1988). Phenotypic modulation of endothelial cells by transforming growth factor-beta depends upon the composition and organization of the extracellular matrix. J Cell Biol.

[280] Belardelli F (1983). Antitumor effects of interferon in mice injected with interferon-sensitive and interferon-resistant Friend leukemia cells. III. Inhibition of growth and necrosis of tumors implanted subcutaneously. Int J Cancer.

[281] Sato N (1990). Actions of TNF and IFN-gamma on angiogenesis in vitro. J Invest Dermatol.

[282] Maheshwari RK (1991). Differential effects of interferon gamma and alpha on in vitro model of angiogenesis. J Cell Physiol.

[283] Fridman WH (2012). The immune contexture in human tumours: impact on clinical outcome. Nat Rev Cancer.

[284] Strieter RM (2005). CXC chemokines in angiogenesis. Cytokine Growth Factor Rev.

[285] Burdick MD (2005). CXCL11 attenuates bleomycin-induced pulmonary fibrosis via inhibition of vascular remodeling. Am J Respir Crit Care Med.

[286] Lasagni L (2003). An alternatively spliced variant of CXCR286 mediates the inhibition of endothelial cell growth induced by IP-10, Mig, and I-TAC, and acts as functional receptor for platelet factor 4. J Exp Med.

[287] Vasudev NS, Reynolds AR (2014). Anti-angiogenic therapy for cancer: current progress, unresolved questions and future directions. Angiogenesis.

[288] de Gramont A (2012). Bevacizumab plus oxaliplatin-based chemotherapy as adjuvant treatment for colon cancer (AVANT): a phase 3 randomised controlled trial. Lancet Oncol.

[289] Yang Y (2016). Discontinuation of anti-VEGF cancer therapy promotes metastasis through a liver revascularization mechanism. Nat Commun.

[290] Haemmerle M (2016). FAK regulates platelet extravasation and tumor growth after antiangiogenic therapy withdrawal. J Clin Invest.

[291] Paez-Ribes M (2009). Antiangiogenic therapy elicits malignant progression of tumors to increased local invasion and distant metastasis. Cancer Cell.

[292] Ebos JM (2009). Accelerated metastasis after short-term treatment with a potent inhibitor of tumor angiogenesis. Cancer Cell.

[293] Wang N, Jain RK, Batchelor TT (2017). New directions in anti-angiogenic therapy for glioblastoma. Neurotherapeutics.

[294] Lucio-Eterovic AK, Piao Y, de Groot JF (2009). Mediators of glioblastoma resistance and invasion during antivascular endothelial growth factor therapy. Clin Cancer Res.

[295] Lu KV (2012). VEGF inhibits tumor cell invasion and mesenchymal transition through a MET/VEGFR2 complex. Cancer Cell.

[296] Jahangiri A (2017). Cross-activating c-Met/beta1 integrin complex drives metastasis and invasive resistance in cancer. Proc Natl Acad Sci USA.

[297] Bergers G, Hanahan D (2008). Modes of resistance to anti-angiogenic therapy. Nat Rev Cancer.

[298] Zarrin B (2017). Acquired tumor resistance to antiangiogenic therapy: mechanisms at a glance. J Res Med Sci.

[299] Liu ZJ, Semenza GL, Zhang HF (2015). Hypoxia-inducible factor 1 and breast cancer metastasis. J Zhejiang Univ Sci B.

[300] Kuczynski EA (2016). Co-option of liver vessels and not sprouting angiogenesis drives acquired sorafenib resistance in hepatocellular carcinoma. J Natl Cancer Inst.

[301] Serova M (2016). Everolimus affects vasculogenic mimicry in renal carcinoma resistant to sunitinib. Oncotarget.

[302] Shaaban S (2016). Targeting bone marrow to potentiate the anti-tumor effect of tyrosine kinase inhibitor in preclinical rat model of human glioblastoma. Int J Cancer Res.

[303] Moccia F (2015). Endothelial progenitor cells support tumour growth and metastatisation: implications for the resistance to anti-angiogenic therapy. Tumour Biol.

[304] Rivera LB (2015). Intratumoral myeloid cells regulate responsiveness and resistance to antiangiogenic therapy. Cell Rep.

[305] Kamba T, McDonald DM (2007). Mechanisms of adverse effects of anti-VEGF therapy for cancer. Br J Cancer.

[306] Carmeliet P, Jain RK (2011). Principles and mechanisms of vessel normalization for cancer and other angiogenic diseases. Nat Rev Drug Discov.

[307] Goel S (2011). Normalization of the vasculature for treatment of cancer and other diseases. Physiol Rev.

[308] Jain RK (2001). Normalizing tumor vasculature with anti-angiogenic therapy: a new paradigm for combination therapy. Nat Med.

[309] Jayson GC, Hicklin DJ, Ellis LM (2012). Antiangiogenic therapy—evolving view based on clinical trial results. Nat Rev Clin Oncol.

[310] Mazzone M (2009). Heterozygous deficiency of PHD2 restores tumor oxygenation and inhibits metastasis via endothelial normalization. Cell.

[311] Sorensen AG (2009). A “vascular normalization index” as potential mechanistic biomarker to predict survival after a single dose of cediranib in recurrent glioblastoma patients. Cancer Res.

[312] Batchelor TT (2013). Improved tumor oxygenation and survival in glioblastoma patients who show increased blood perfusion after cediranib and chemoradiation. Proc Natl Acad Sci USA.

[313] Sorensen AG (2012). Increased survival of glioblastoma patients who respond to antiangiogenic therapy with elevated blood perfusion. Cancer Res.

[314] Shrimali RK (2010). Antiangiogenic agents can increase lymphocyte infiltration into tumor and enhance the effectiveness of adoptive immunotherapy of cancer. Cancer Res.

[315] Yang J, Yan J, Liu B (2018). Targeting VEGF/VEGFR to modulate antitumor immunity. Front Immunol.

[316] Xin H (2009). Sunitinib inhibition of Stat3 induces renal cell carcinoma tumor cell apoptosis and reduces immunosuppressive cells. Cancer Res.

[317] Adotevi O (2010). A decrease of regulatory T cells correlates with overall survival after sunitinib-based antiangiogenic therapy in metastatic renal cancer patients. J Immunother.

[318] Martino EC (2016). Immune-modulating effects of bevacizumab in metastatic non-small-cell lung cancer patients. Cell Death Discov.

[319] Olsson AK (2014). Therapeutic vaccination targeting the tumour vasculature. Biochem Soc Trans.

[320] Huijbers EJ (2010). Vaccination against the extra domain-B of fibronectin as a novel tumor therapy. FASEB J.

[321] Femel J (2014). Therapeutic vaccination against fibronectin ED-A attenuates progression of metastatic breast cancer. Oncotarget.

[322] Chaudhary A (2012). TEM8/ANTXR1 blockade inhibits pathological angiogenesis and potentiates tumoricidal responses against multiple cancer types. Cancer Cell.

[323] Szot C (2018). Tumor stroma-targeted antibody-drug conjugate triggers localized anticancer drug release. J Clin Invest.

[324] Byrd TT (2018). TEM8/ANTXR1-specific CAR T cells as a targeted therapy for triple-negative breast cancer. Cancer Res.

[325] Hamzah J (2008). Vascular targeting of anti-CD40 antibodies and IL-2 into autochthonous tumors enhances immunotherapy in mice. J Clin Invest.

[326] Johansson A (2012). Tumor-targeted TNFalpha stabilizes tumor vessels and enhances active immunotherapy. Proc Natl Acad Sci USA.

[327] Khan KA, Kerbel RS (2018). Improving immunotherapy outcomes with anti-angiogenic treatments and vice versa. Nat Rev Clin Oncol.

[328] Georganaki M, van Hooren L, Dimberg A (2018). Vascular targeting to increase the efficiency of immune checkpoint blockade in cancer. Front Immunol.

[329] Hayasaka H (2010). Neogenesis and development of the high endothelial venules that mediate lymphocyte trafficking. Cancer Sci.

[330] Hindley JP (2012). T-cell trafficking facilitated by high endothelial venules is required for tumor control after regulatory T-cell depletion. Cancer Res.

[331] Colbeck EJ (2017). Treg depletion licenses T cell-driven HEV neogenesis and promotes tumor destruction. Cancer Immunol Res.

[332] Allen E (2017). Combined antiangiogenic and anti-PD-L1 therapy stimulates tumor immunity through HEV formation. Sci Transl Med.

[333] He B (2018). Vascular targeting of LIGHT normalizes blood vessels in primary brain cancer and induces intratumoural high endothelial venules. J Pathol.

[334] Patel MM, Patel BM (2017). Crossing the blood-brain barrier: recent advances in drug delivery to the brain. CNS Drugs.

[335] Sandler A (2006). Paclitaxel-carboplatin alone or with bevacizumab for non-small-cell lung cancer. N Engl J Med.

[336] Tewari KS (2017). Bevacizumab for advanced cervical cancer: final overall survival and adverse event analysis of a randomised, controlled, open-label, phase 3 trial (Gynecologic Oncology Group 240). Lancet.

[337] Perren TJ (2011). A phase 3 trial of bevacizumab in ovarian cancer. N Engl J Med.

[338] Escudier B (2007). Bevacizumab plus interferon alfa-2a for treatment of metastatic renal cell carcinoma: a randomised, double-blind phase III trial. Lancet.

[339] Wick W (2017). Lomustine and bevacizumab in progressive glioblastoma. N Engl J Med.

[340] Fuchs CS (2014). Ramucirumab monotherapy for previously treated advanced gastric or gastro-oesophageal junction adenocarcinoma (REGARD): an international, randomised, multicentre, placebo-controlled, phase 3 trial. Lancet.

[341] Tabernero J (2015). Ramucirumab versus placebo in combination with second-line FOLFIRI in patients with metastatic colorectal carcinoma that progressed during or after first-line therapy with bevacizumab, oxaliplatin, and a fluoropyrimidine (RAISE): a randomised, double-blind, multicentre, phase 3 study. Lancet Oncol.

[342] Zhu AX (2019). Ramucirumab after sorafenib in patients with advanced hepatocellular carcinoma and increased alpha-fetoprotein concentrations (REACH-2): a randomised, double-blind, placebo-controlled, phase 3 trial. Lancet Oncol.

[343] Garon EB (2014). Ramucirumab plus docetaxel versus placebo plus docetaxel for second-line treatment of stage IV non-small-cell lung cancer after disease progression on platinum-based therapy (REVEL): a multicentre, double-blind, randomised phase 3 trial. Lancet.

[344] Bonner JA (2006). Radiotherapy plus cetuximab for squamous-cell carcinoma of the head and neck. N Engl J Med.

[345] Heinemann V (2014). FOLFIRI plus cetuximab versus FOLFIRI plus bevacizumab as first-line treatment for patients with metastatic colorectal cancer (FIRE-3): a randomised, open-label, phase 3 trial. Lancet Oncol.

[346] Price TJ (2014). Panitumumab versus cetuximab in patients with chemotherapy-refractory wild-type KRAS exon 2 metastatic colorectal cancer (ASPECCT): a randomised, multicentre, open-label, non-inferiority phase 3 study. Lancet Oncol.

[347] Thatcher N (2015). Necitumumab plus gemcitabine and cisplatin versus gemcitabine and cisplatin alone as first-line therapy in patients with stage IV squamous non-small-cell lung cancer (SQUIRE): an open-label, randomised, controlled phase 3 trial. Lancet Oncol.

[348] Gianni L (2014). Neoadjuvant and adjuvant trastuzumab in patients with HER2-positive locally advanced breast cancer (NOAH): follow-up of a randomised controlled superiority trial with a parallel HER2-negative cohort. Lancet Oncol.

[349] Bang YJ (2010). Trastuzumab in combination with chemotherapy versus chemotherapy alone for treatment of HER2-positive advanced gastric or gastro-oesophageal junction cancer (ToGA): a phase 3, open-label, randomised controlled trial. Lancet.

[350] Hurvitz SA (2018). Neoadjuvant trastuzumab, pertuzumab, and chemotherapy versus trastuzumab emtansine plus pertuzumab in patients with HER2-positive breast cancer (KRISTINE): a randomised, open-label, multicentre, phase 3 trial. Lancet Oncol.

[351] Llovet JM (2008). Sorafenib in advanced hepatocellular carcinoma. N Engl J Med.

[352] Escudier B (2007). Sorafenib in advanced clear-cell renal-cell carcinoma. N Engl J Med.

[353] Brose MS (2014). Sorafenib in radioactive iodine-refractory, locally advanced or metastatic differentiated thyroid cancer: a randomised, double-blind, phase 3 trial. Lancet.

[354] Demetri GD (2006). Efficacy and safety of sunitinib in patients with advanced gastrointestinal stromal tumour after failure of imatinib: a randomised controlled trial. Lancet.

[355] Raymond E (2011). Sunitinib malate for the treatment of pancreatic neuroendocrine tumors. N Engl J Med.

[356] Motzer RJ (2009). Overall survival and updated results for sunitinib compared with interferon alfa in patients with metastatic renal cell carcinoma. J Clin Oncol.

[357] Dematteo RP (2009). Adjuvant imatinib mesylate after resection of localised, primary gastrointestinal stromal tumour: a randomised, double-blind, placebo-controlled trial. Lancet.

[358] Druker BJ (2006). Five-year follow-up of patients receiving imatinib for chronic myeloid leukemia. N Engl J Med.

[359] Fielding AK (2014). UKALLXII/ECOG2993: addition of imatinib to a standard treatment regimen enhances long-term outcomes in Philadelphia positive acute lymphoblastic leukemia. Blood.

[360] Motzer RJ (2013). Pazopanib versus sunitinib in metastatic renal-cell carcinoma. N Engl J Med.

[361] van der Graaf WT (2012). Pazopanib for metastatic soft-tissue sarcoma (PALETTE): a randomised, double-blind, placebo-controlled phase 3 trial. Lancet.

[362] Kim ES (2008). Gefitinib versus docetaxel in previously treated non-small-cell lung cancer (INTEREST): a randomised phase III trial. Lancet.

[363] Lee SM (2012). First-line erlotinib in patients with advanced non-small-cell lung cancer unsuitable for chemotherapy (TOPICAL): a double-blind, placebo-controlled, phase 3 trial. Lancet Oncol.

[364] Moore MJ (2007). Erlotinib plus gemcitabine compared with gemcitabine alone in patients with advanced pancreatic cancer: a phase III trial of the National Cancer Institute of Canada Clinical Trials Group. J Clin Oncol.

[365] Wells SA (2012). Vandetanib in patients with locally advanced or metastatic medullary thyroid cancer: a randomized, double-blind phase III trial. J Clin Oncol.

[366] Li J (2015). Regorafenib plus best supportive care versus placebo plus best supportive care in Asian patients with previously treated metastatic colorectal cancer (CONCUR): a randomised, double-blind, placebo-controlled, phase 3 trial. Lancet Oncol.

[367] Demetri GD (2013). Efficacy and safety of regorafenib for advanced gastrointestinal stromal tumours after failure of imatinib and sunitinib (GRID): an international, multicentre, randomised, placebo-controlled, phase 3 trial. Lancet.

[368] Bruix J (2017). Regorafenib for patients with hepatocellular carcinoma who progressed on sorafenib treatment (RESORCE): a randomised, double-blind, placebo-controlled, phase 3 trial. Lancet.

[369] Martin M (2017). Neratinib after trastuzumab-based adjuvant therapy in HER2-positive breast cancer (ExteNET): 5-year analysis of a randomised, double-blind, placebo-controlled, phase 3 trial. Lancet Oncol.

[370] Baselga J (2012). Lapatinib with trastuzumab for HER2-positive early breast cancer (NeoALTTO): a randomised, open-label, multicentre, phase 3 trial. Lancet.

[371] Sequist LV (2013). Phase III study of afatinib or cisplatin plus pemetrexed in patients with metastatic lung adenocarcinoma with EGFR mutations. J Clin Oncol.

[372] Rini BI (2011). Comparative effectiveness of axitinib versus sorafenib in advanced renal cell carcinoma (AXIS): a randomised phase 3 trial. Lancet.

[373] Abou-Alfa GK (2018). Cabozantinib in Patients with Advanced and Progressing Hepatocellular Carcinoma. N Engl J Med.

[374] Elisei R (2013). Cabozantinib in progressive medullary thyroid cancer. J Clin Oncol.

[375] Powles T (2018). Outcomes based on prior therapy in the phase 3 METEOR trial of cabozantinib versus everolimus in advanced renal cell carcinoma. Br J Cancer.

[376] Kudo M (2018). Lenvatinib versus sorafenib in first-line treatment of patients with unresectable hepatocellular carcinoma: a randomised phase 3 non-inferiority trial. Lancet.

[377] Schlumberger M (2015). Lenvatinib versus placebo in radioiodine-refractory thyroid cancer. N Engl J Med.

[378] Tabernero J (2014). Aflibercept versus placebo in combination with fluorouracil, leucovorin and irinotecan in the treatment of previously treated metastatic colorectal cancer: prespecified subgroup analyses from the VELOUR trial. Eur J Cancer.

[379] Rajkumar SV (2002). Combination therapy with thalidomide plus dexamethasone for newly diagnosed myeloma. J Clin Oncol.

[380] Rajkumar SV (2010). Lenalidomide plus high-dose dexamethasone versus lenalidomide plus low-dose dexamethasone as initial therapy for newly diagnosed multiple myeloma: an open-label randomised controlled trial. Lancet Oncol.

[381] Motzer RJ (2008). Efficacy of everolimus in advanced renal cell carcinoma: a double-blind, randomised, placebo-controlled phase III trial. Lancet.

[382] Baselga J (2012). Everolimus in postmenopausal hormone-receptor-positive advanced breast cancer. N Engl J Med.

[383] Yao JC (2011). Everolimus for advanced pancreatic neuroendocrine tumors. N Engl J Med.

[384] Pavel ME (2017). Health-related quality of life for everolimus versus placebo in patients with advanced, non-functional, well-differentiated gastrointestinal or lung neuroendocrine tumours (RADIANT-4): a multicentre, randomised, double-blind, placebo-controlled, phase 3 trial. Lancet Oncol.

[385] Franz DN (2013). Efficacy and safety of everolimus for subependymal giant cell astrocytomas associated with tuberous sclerosis complex (EXIST-1): a multicentre, randomised, placebo-controlled phase 3 trial. Lancet.

